# Genome characterization of two bile-isolated *Vibrio fluvialis* strains: an insight into pathogenicity and bile salt adaption

**DOI:** 10.1038/s41598-017-12304-8

**Published:** 2017-09-19

**Authors:** Beiwen Zheng, Xiawei Jiang, Hong Cheng, Lihua Guo, Jing Zhang, Hao Xu, Xiao Yu, Chen Huang, Jinru Ji, Chaoqun Ying, Youjun Feng, Yonghong Xiao, Lanjuan Li

**Affiliations:** 1 State Key Laboratory for Diagnosis and Treatment of Infectious Disease, Collaborative Innovation Center for Diagnosis and Treatment of Infectious Diseases, The First Affiliated Hospital, College of Medicine, Zhejiang University, Hangzhou, China; 2 College of Basic Medical Sciences, Zhejiang Chinese Medical University, Hangzhou, China; 3 Key Laboratory of Marine Ecosystem and Biogeochemistry, Second Institute of Oceanography, State Oceanic Administration, Hangzhou, China; 4 Department of Respiratory Diseases, The First Affiliated Hospital, College of Medicine, Zhejiang University, Hangzhou, China; 5 Department of Medical Microbiology and Parasitology, Zhejiang University School of Medicine, Hangzhou, China

## Abstract

*Vibrio fluvialis* is recognized as an emerging pathogen. However, not much is known about the mechanism of its pathogenesis, and its adaptation to a special niche such as the gall bladder. Here we describe two *V*. *fluvialis* strains that cause acute cholecystitis. It is noteworthy that both strains were susceptible to all antibiotics tested, which is in contrast to previous studies, suggesting substantial genetic diversity among *V*. *fluvialis* isolates. In agreement with their survival and growth in the gall bladder, the genomes of strains 12605 and 3663 contain a considerable number of genes that confer resistance to bile, including *toxR*, *omp* *U*, *tolC*, *cmeABC*, *rlpB*, *yrbK*, *rpoS*, *damX* and *gltK*. Furthermore, integrative and conjugative elements (ICEs), virulence factors and prophage regions were also detected in strains 12605 and 3663, reflecting their flexibility in recombination during the evolution of pathogenicity. Comparative analysis of nine available genomes of *V*. *fluvialis* revealed a core genome consisting of 3,147 genes. Our results highlight the association of *V*. *fluvialis* with a rare disease profile and shed light on the evolution of pathogenesis and niche adaptation of *V*. *fluvialis*.

## Introduction

*Vibrio fluvialis* is a halophilic Gram-negative bacterium, which is considered to be an emerging pathogen, found mostly in aquatic environments^1^. *V*. *fluvialis* infection is mainly associated with sporadic cases and outbreaks of gastroenteritis with cholera-like diarrhea^1^. Although rare, *V*. *fluvialis* can also cause extraintestinal infections, including hemorrhagic cellulitis with cerebritis^2^, bacteremia^3^, peritonitis^4^, acute otitis^5^ and endophthalmitis^6^. To date, only two reports have described biliary tract infection caused by this bacterium^7,8^. The route of *V*. *fluvialis* entry into the biliary system remains unknown, and it was assumed to be via a cutaneous lesion or due to gastrointestinal translocation upon consumption of contaminated seafood as observed in other *Vibrio* infections^8^.

Molecular characterization of this pathogen is particularly important, owing to the increasing appearance of multidrug resistant strains and their potential to cause epidemics^9^. Several studies have indicated that Chinese hamster ovary (CHO) cell elongation factor, enterotoxin, lipase, protease, cytotoxin, hemolysin and quorum sensing systems contribute to the pathogenicity of this organism^1^. Whole-genome sequences of several *V*. *fluvialis* stains have been described earlier, including two strains that cause diarrhea in human and two environmental strains^10,11^. These genome data help in understanding the genetic basis of the physiology, biochemical pathways and evolution of *V*. *fluvialis*. However, the mechanisms of pathogenesis and its adaptation to a special niche, such as gallbladder, are yet to be explored.

Here we describe the isolation and characterization of two *V*. *fluvialis* strains that cause acute cholecystitis. The complete genome sequence of strain 12605 and the draft genome sequence of strain 3663 were obtained by whole genome sequencing. Genes involved in bile resistance were identified in these genomes. Integrative and conjugative elements (ICEs), virulence factors and prophage regions were also found in both genomes. Comparative genomics analysis was conducted with nine *V*. *fluvialis* strains. Our findings will contribute to the understanding of the phylogenetic diversity and niche adaptation of the two strains, while also shedding light on the evolution of pathogenesis in *V*. *fluvialis*.

## Results

### Case record

#### Case 1

A 76-year-old male was admitted to our hospital with fever, vomiting and a sudden upper abdominal pain on July 2014. He reported that the recurring pain suddenly began seven days ago. Lower back pain, chills, vomiting and diarrhea were not documented during the evolution of symptoms. The medical history was remarkable, as he had a history of gallstones and had undergone surgical treatment of cholangiocarcinoma two weeks prior. Ultrasound of the biliary tract revealed a thickened gallbladder wall and pericholecystic fluid, with no visible stones, an observation consistent with the presence of acute cholecystitis. Empirical parenteral administration of 500 mg of levofloxacin every 8 h was started on the first day of hospitalization. On the third day, a curved rod shaped Gram-negative bacterium, designated as 3663, was isolated from the bile culture. The fever, abdominal pain and the clinical condition improved after antibiotic treatment. The patient was discharged two weeks later and prescribed oral cephalosporin antibiotics.

#### Case 2

A 55-year-old man presented at an emergency department in 2013 with a four-day history of right upper quadrant pain and vomiting. He lived in the coast of Hangzhou Gulf and denied having any history of travel, but had high potential exposure to seafood. He had a history of gallstones. Abdominal computed tomography clearly showed a distended gallbladder with thickened wall and gallstones. The patient was hospitalized with a presumptive diagnosis of acute calculous cholecystitis and received cefotiam treatment. The bile cultures yielded significant growth of a curved rod shaped Gram-negative bacterium (designated as 12605). The patient was treated with intravenous fluids and broad-spectrum antibiotics for five days. His condition improved clinically, with resolution of the abdominal pain and normalization of his laboratory and ultrasonography findings. He was discharged from the hospital with a 10-day course of treatment and recovered fully.

### Phenotypic and genotypic characterization of clinical isolates

MALDI-TOF analysis indicated that the strains 12605 and 3663 are highly similar to *V*. *fluvialis* from the default Bruker database (matching scores of >2.0). 16 S rRNA sequences of 12605 and 3663 were aligned by BLAST against the latest version of EzBioCloud’s database^12^ and the results showed that both strains share 99.86% identity with *V*. *fluvialis* NBRC 103150^T^. Growth curves of two *V*. *fluvialis* isolates demonstrated that bile resistance was observed in both isolates (see Supplementary Fig. S1). Antimicrobial susceptibility tests demonstrated that both strains were susceptible to all antibiotics tested (see Supplementary Table S1).

### General genome features

The genomes of 12605 and 3663 were sequenced as described in material and methods. Sequencing of strain 12605 revealed the presence of two complete chromosomes (one chromosome is 3,171,566 bp in length and the other is 1,680,098 bp in length) with an average G+C content of 50.1%. The two chromosomes contain 4,395 protein coding genes, 113 tRNAs and 37 rRNAs. Sequencing of strain 3663 revealed a genome size of 4,849,960 bp with a G+C content of 49.9% (207 contigs). These contigs contain 4,441 protein coding genes, 87 tRNAs and 12 rRNAs. The genomic features of the strains 12605 and 3663, and eight other reference *V*. *fluvialis* strains are summarized in Table 1.

**Table 1:** Global features of the *V*. *fluvialis* strains.

Strain	Isolation source	Host	Size (Mb)	GC%	Accession no.	Coding sequence	rRNA	tRNA
12605	bile	*Homo sapiens*	4.85	50.1	CP019118, CP019119	4395	37	113
3663	bile	*Homo sapiens*	4.85	49.9	JXXQ01	4441	12	87
ATCC 33809	feces	*Homo sapiens*	4.83	49.9	CP014034, CP014035	4406	31	108
PG41	patient with severe diarrhea	*Homo sapiens*	5.34	48.1	ASXS01	4845	4	122
I21563	patient with severe diarrhea	*Homo sapiens*	4.37	50.1	ASXT01	4050	7	65
560	—	marine oysters	4.66	50.1	JQHW01	4364	5	92
539	—	marine oysters	4.99	50	JQHX01	5609	6	87
S1110	seawater	—	4.52	50	LKHR01	4129	4	48
NBRC 103150	human feces	*Homo sapiens*	4.75	49.9	BCZR01	4373	4	64

### Phylogenetic analysis of strains 12605 and 3663

**Figure 1:**
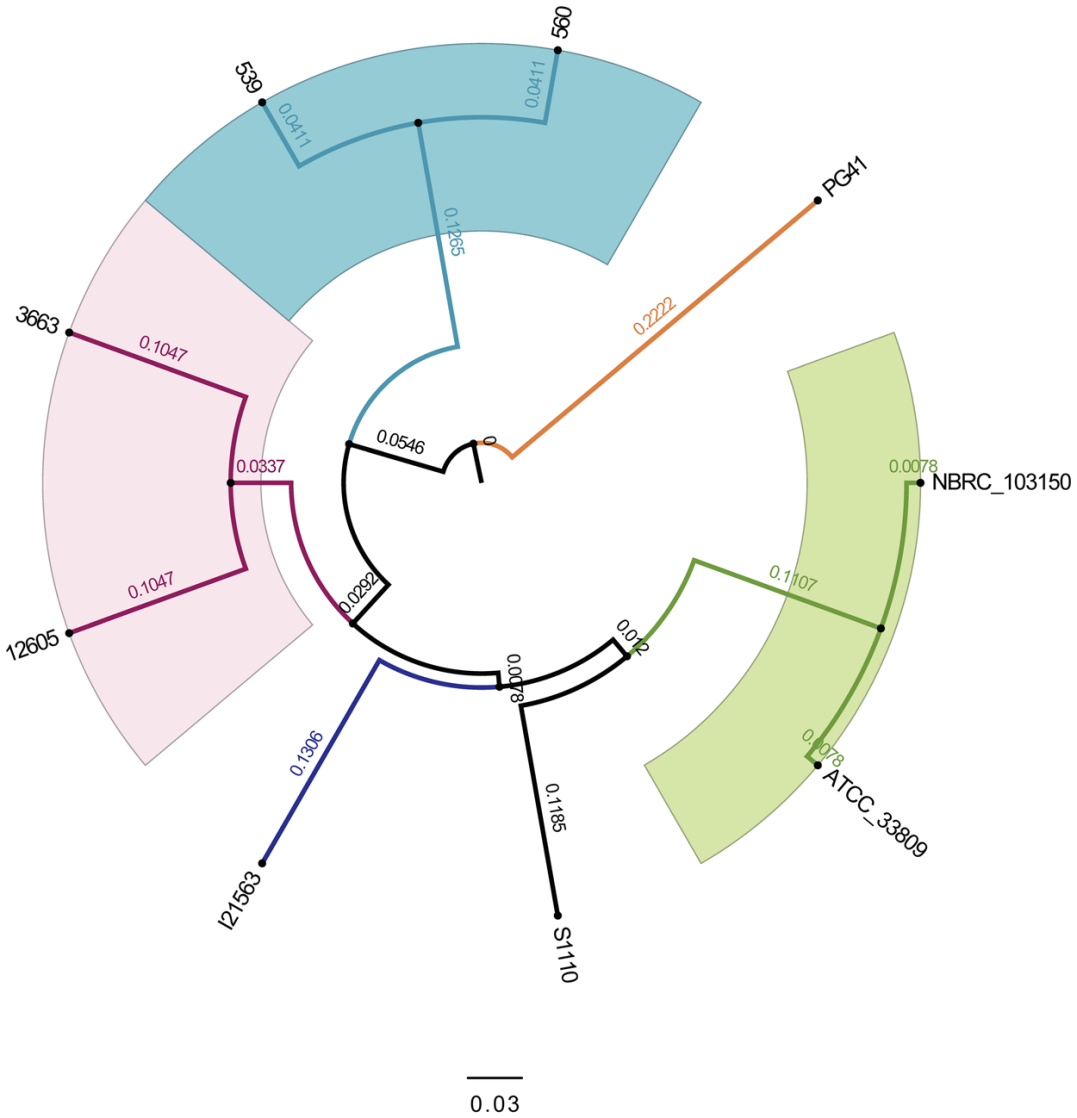
Dendrogram of *V*. *fluvialis* strains based on genomic BLAST. The genomic BLAST file was downloaded from the NCBI database and the tree was visualized by FigTree v1.4 ^42^.

To further understand the phylogenetic relationship between the *V*. *fluvialis* strains 12605 and 3663 and other strains within this species, their genome sequences were obtained from the NCBI database and analyzed. To date, ten genomes of *V*. *fluvialis* have been deposited in the NCBI database, including strains 12605 and 3663. To obtain an estimate of the overall similarity between the ten *V*. *fluvialis* genomes, we calculated their average nucleotide identity (ANI). To our surprise, strain NCTC 11327 showed remarkably low ANI values as compared to all other strains (73.4–73.5%) (see Supplementary Fig. S2). As recommended by several early studies, ANI values of about 95–96% are considered to be the species boundary^13–15^, indicating that strain NCTC 11327 may not belong to *V*. *fluvialis* species. To further test this assumption, we compared the 16S rRNA gene of NCTC 11327 against the NCBI nr database^16,17^. The results showed it shared 100% sequence identity with *V*. *vulnificus* strain CAPL-B-VVF2 (KX904714.1). Thus, the genome sequence (accession no. LMTE00000000.1) deposited under the name of *V*. *fluvialis* strain NCTC 11327 is actually a member of *V*. *vulnificus* species; this genome was thus excluded from our analysis. The ANI was then recalculated among the remaining nine genomes, and the values among them were found to range between 97.1–100%, indicating a close relationship with each other (see Supplementary Fig. S3). The highest similarity with an ANI value of 100% was found between the strains ATCC 33809 and NBRC 103150. By searching against the NITE Biological Resource Center (NBRC) database, we found that NBRC 103150 is actually ATCC 33809. Therefore, the ANI value of 100% between these two genomes confirms that they actually belong to the same strain that were deposited in two different culture centers. In order to provide a high-resolution view of phylogeny, a phylogenetic tree was constructed based on the genome alignments of the nine *V*. *fluvialis* strains (Fig. 1). As expected, strains 12605 and 3663, both isolated from bile, fell into the same clade.

### Identification of Integrative and Conjugative Elements (ICEs)

**Figure 2:**
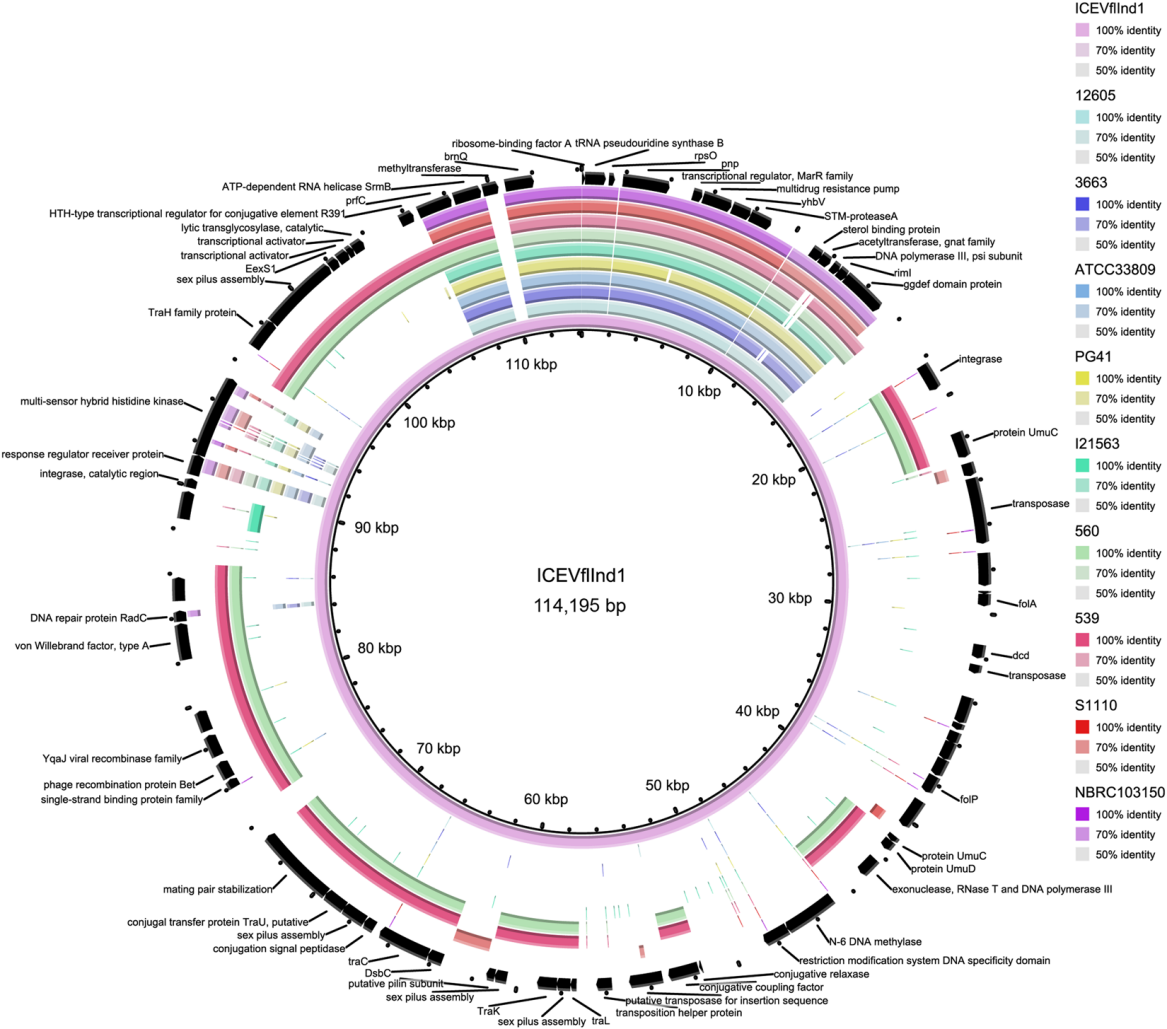
Comparison of integrative and conjugative elements ICE*Vfl*Ind1 and *V*. *fluvialis* genomes. Genes denoted by arrows are based on the annotation of ICE*Vfl*Ind1 (accession number of GQ463144).

Integrative and conjugative elements (ICEs) are a diverse group of mobile elements found in bacteria^18^ that enable horizontal gene transfer (HGT) of virulence genes, antibiotic resistance genes, etc.^19^ To identify ICEs in our two bile-isolated strains (12605 and 3663), we compared their genomes against the ICEberg database^19^ by BLAST-2.3.0+ ^20^ program. Many genes belonging to various types of ICEs were shortlisted (see Supplementary Tables S2 and S3). A total of 15 genes belong to ICE*Vfl*Ind1 (ICEberg ID 36) were found in both strains. To identify whether the genes of ICE*Vfl*Ind1 existed in all *V*. *fluvialis* strains, we compared the complete sequences of ICE*Vfl*Ind1 from GenBank database (GQ463144) with the whole genomes of the nine strains, as shown in Fig. 2. The strains carried most of genes of ICE*Vfl*Ind1, with strains 539 and 560 carrying more genes than other the strains.

### Identification of virulence-associated genes

Further screening of the genomes for putative virulence-associated genes was conducted by aligning ORF encoded protein sequences to the virulence factor database (VFDB). Both genomes were found to contain many putative virulence factors (see Supplementary Tables S4 and S5). In addition, multiple copies of heat shock proteins including GroES, GroEL, and HspA homologs were found in both genomes and these proteins were demonstrated to be essential for bacterial survival and association for colonization in *Helicobacter pylori* ^21^. Consistent with a previous study, homologs of flagellar biosynthetic proteins (FliD, FliE, FliF, FliG, FliH, FliI, FliJ, FliK, FliL, FliM, FliN, FliO, FliP, FliQ, FliR, and FliS) were also found in these genomes^22^. Furthermore, we searched the genomes of 12605 and 3663 for prophage regions and found that both strains harbored several prophage regions (see Supplementary Table S6). The phage-like sequences are believed to improve cell adhesion and ability to acquire antibiotic resistance that can enable bacteria to survive in new environments and become pathogens^23^.

### Identification of putative bile resistance-related genes

It has been demonstrated that bile salts have potent antimicrobial properties that can cause damage to bacteria^24^. For survival in bile and cause cholecystitis in humans, bacteria have to overcome this barrier. In response to bile salt exposure, bacteria can induce efflux systems, induce elevated resistance to bile toxicity, enhance motility, remodel outer membrane proteins, and even promote biofilm formation^25^. ToxR, which is a membrane-associated transcription factor, was found to play a major role in bile resistance of *V*. *fluvialis* and other *Vibrio* species^26^. Another protein GltK, which is a glutamate/aspartate transport system permease protein, was also shown to be involved in bile resistance^27^. Analysis of the genomes of strains 12605 and 3663 revealed the existence of genes encoding ToxR and GltK in both genomes. Furthermore, a considerable number of putative bile resistance-related proteins such as DamX (an inner membrane protein involved in bile resistance), lipopolysaccharide transporters, membrane transport systems, and polymerase sigma factor were encoded in the genomes of both 12605 and 3663. These genes may play important roles in the survival of 12605 and 3663 in the gall bladder.

### Comparative genomic analysis of *V*. *fluvialis* strains

**Figure 3:**
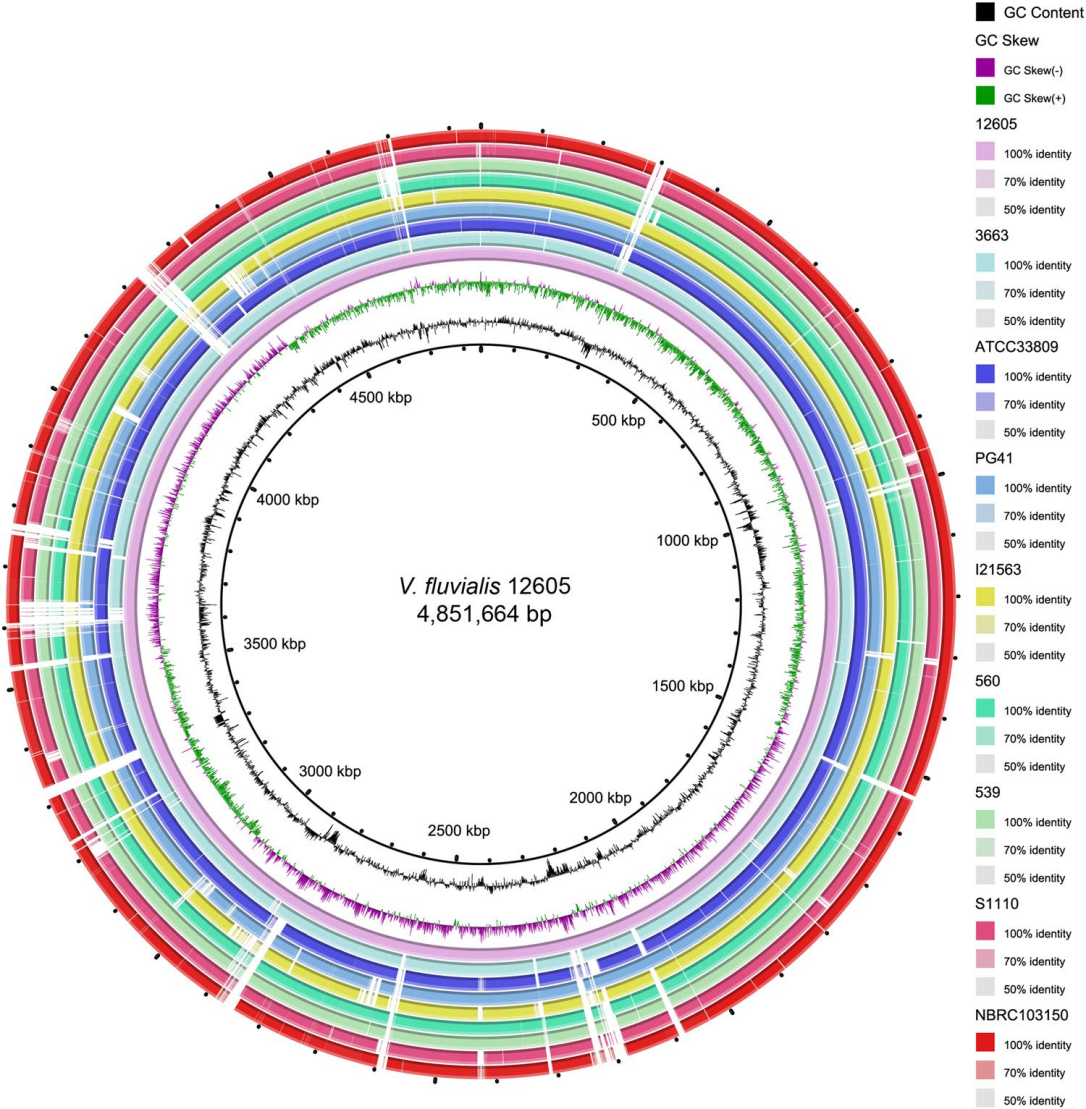
BLAST atlas of the genomes of various *V*. *fluvialis* strains. The circles from inside to outside: GC content, and GC skew of *V*. *fluvialis* 12605; BLASTN pairwise comparison of the *V*. *fluvialis* genomes: 12605, 3663, ATCC 33809, PG41, I21563, 560 539, S1110 and NBRC 103150. The white and colored regions of the outer rings indicate regions absent and present, respectively.

**Figure 4:**
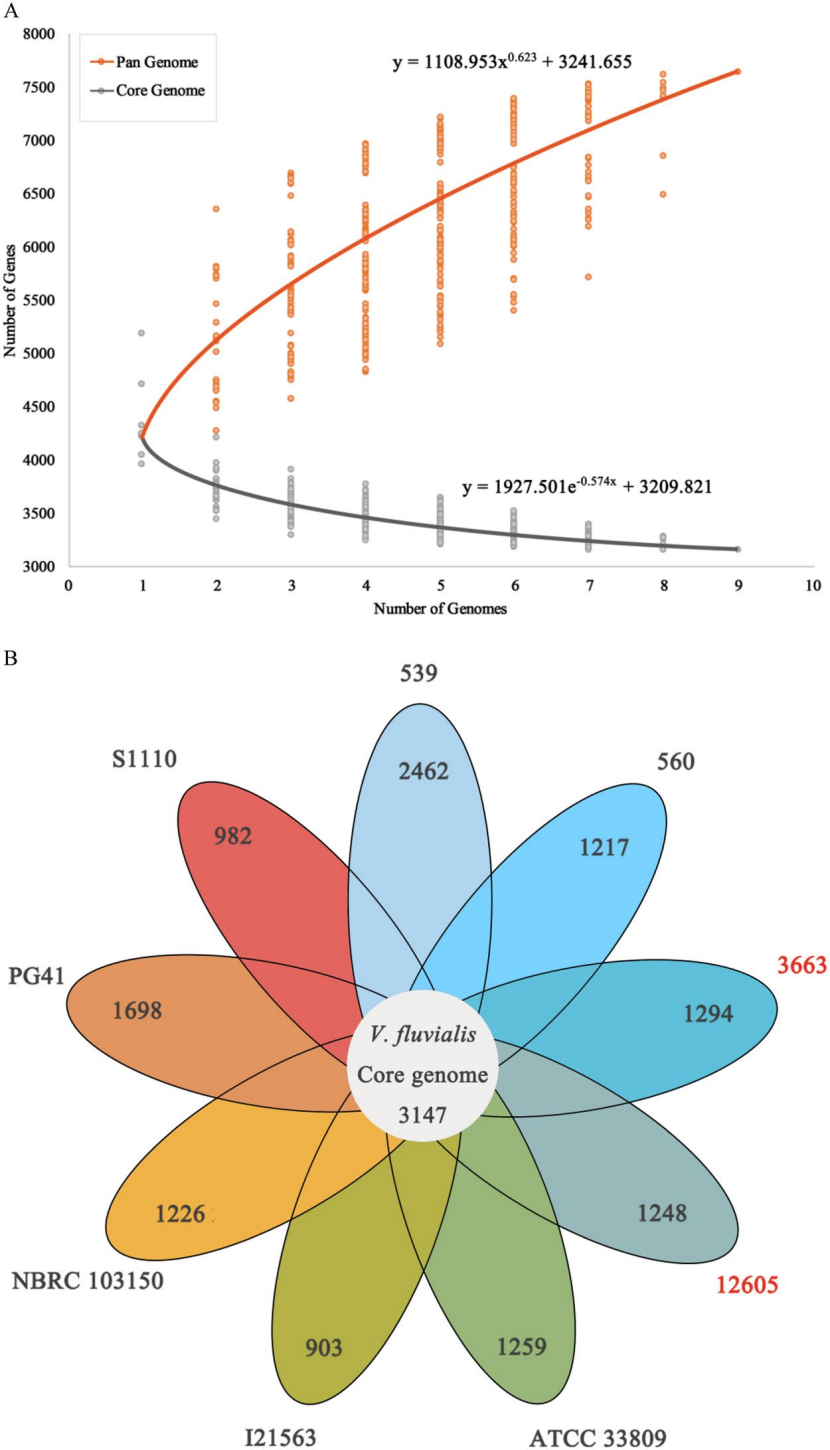
Comparative genomic analysis of *V*.*fluvialis* strains. (**A**)Pan-genome and core genome profiles, the numbers of new genes in the *V*. *fluvialis* pan-genome and core genome are plotted against the number of genomes added; (**B**) Venn diagram showing the number of species-specific gene families in the genome of each strain. The number of core genomes is represented in the center.

The circular maps and BLAST visualization of the nine *V*. *fluvialis* genomes are illustrated in Fig. 3. The results were in agreement with our phylogenetic tree. Pan-genome analysis of the nine genomes revealed that the core genome consists of 3,147 genes from all 40,602 total genes, which constituted the pan-genome. The pan-genome size of these nine strains was 7,625 and their increased tendency showed the pan-genome could be taken as an open pan-genome. (Fig. 4). The ratio of core-genome in each species ranged from 22% to 44% and was correlated with the total coding sequence (CDS) numbers (see Supplementary Table S7). The ratio of core/pan-genome with nine strains was 41.3%. We also analyzed the functional classifications of ortholog clusters using the COG database. The results obtained are summarized in Table 2. With the exception of poorly characterized or uncharacterized COGs, the most abundant category in the pan genome was [K] transcription. The next abundant COGs were [E] Amino acid transport and metabolism, followed by [T] Signal transduction mechanisms. In the core genome, [E] Amino acid transport and metabolism was the largest gene family, followed by [K] transcription and [T] Signal transduction mechanisms. Subsequently, the proportion of the conserved groups (core, dispensable, and specific genomes) in each category was investigated further (see Supplementary Fig. S4).

**Table 2:** COG distribution in the pan genome of nine *V*. *fluvialis* strains.

COG category	Core	Specific	Dispensable	Total
INFORMATION STORAGE AND PROCESSING	532	282	229	1043
[J] Translation, ribosomal structure and biogenesis	170	33	25	228
[A] RNA processing and modification	1	0	0	1
[K] Transcription	253	100	120	473
[L] Replication, recombination and repair	107	149	84	340
[B] Chromatin structure and dynamics	1	0	0	1
CELLULAR PROCESSES AND SIGNALING	781	409	337	1527
[D] Cell cycle control, cell division, chromosome partitioning	27	17	8	52
[Y] Nuclear structure	0	0	0	0
[V] Defense mechanisms	37	52	46	135
[T] Signal transduction mechanisms	228	120	94	442
[M] Cell wall/membrane/envelope biogenesis	137	89	90	316
[N] Cell motility	124	43	43	210
[Z] Cytoskeleton	0	0	0	0
[W] Extracellular structures	0	0	0	0
[U] Intracellular trafficking, secretion, and vesicular transport	98	44	30	172
[O] Posttranslational modification, protein turnover, chaperones	130	44	26	200
METABOLISM	1172	486	398	2056
[C] Energy production and conversion	172	88	72	332
[G] Carbohydrate transport and metabolism	177	104	116	397
[E] Amino acid transport and metabolism	278	99	71	448
[F] Nucleotide transport and metabolism	84	28	13	125
[H] Coenzyme transport and metabolism	140	57	34	231
[I] Lipid transport and metabolism	87	26	27	140
[P] Inorganic ion transport and metabolism	168	63	52	283
[Q] Secondary metabolites biosynthesis, transport and catabolism	66	21	13	100
POORLY CHARACTERIZED	1029	1770	823	3622
[R] General function prediction only	366	192	126	684
[S] Function unknown	280	81	85	446
[-] Unclassified	383	1497	612	2492

## Discussion

Though the emerging human pathogen *V*. *fluvialis* was known to cause diarrheal illness and extraintestinal infections for quite some time, in depth studies on the pathogenesis of *V*. *fluvialis* have not been performed so far^22^. To our knowledge, there are only two known cases of *V*. *fluvialis*-associated biliary tract infections and no reports of acute cholecystitis since its first description in human infection. Very little is known about the clinical importance of *V*. *fluvialis* in biliary tract infection and its niche adaptation to bile salts. In this study, we report two cases of acute cholecystitis. Since only *V*. *fluvialis* was detected from the bile samples of the affected individuals, we conclude that the inflammatory responses were caused by *V*. *fluvialis* infection. However, the source of infection is difficult to trace. We speculate that the patients were brought in contact with pathogens in an aquatic environment, since *V*. *fluvialis* occurs widely in the aquatic realm and our patients may have potentially been exposed to seafood and aquatic environment^1^.

Identification of the specific species is essential for appropriate antimicrobial treatment and improved clinical care. However, this remains a major challenge with the API20E and Vitek 2 systems due to similarities in the phenotypic characteristics of *V*. *fluvialis* and other *Vibrio* species^1^. In the current study, we employed MALDI-TOF and 16 S rRNA sequencing methods for rapid identification of *V*. *fluvialis*, which confirmed the combined strategy as a reliable method for accurate identification.

In this study, the MICs of all antibiotics tested were found to be within the susceptible range. This finding may explain the improved recovery of two patients following treatment with antibiotics. In contrast, Liang *et al*. reported that most of the *V*. *fluvialis* strains from China were resistant to β-lactams, azithromycin, and sulfamethoxazole, and that the clinical isolates showed higher resistance in general than the environmental isolates^9^.

To further ascertain the adaptive features and essential genes of *V*. *fluviali*s 12605 and 3663 for survival in the gallbladder, we performed WGS and comparative genomic analysis. Bile salts can damage membrane lipids and cause misfolding and denaturation of intracellular proteins^28^. Therefore, bacteria must survive in high concentrations of bile salts before invading the gall bladder epithelial cells. In agreement with their survival and growth in the gall bladder, the genomes of 12605 and 3663 were found to encode several bile resistance-related genes. These include *toxR, ompU*, *ompT*, *tolC*, which are essential for bile resistance in *V*. *cholera* ^26,29^; *cmeABC*, which mediates enhanced resistance to bile in *Campylobacter* ^30^; *gltK*, which has been confirmed to be involved in bile resistance in *Enterococcus faecium* ^27^; *rlpB, yrbK* and *rpoS*, which have been reported to be involved in bile resistance in *Salmonella* ^31^; and *damX*, which encodes an inner membrane protein involved in bile resistance in *S*. *enterica* ^32^.

Phage-mediated horizontal gene transfer is known to drive virulence and genomic diversification of bacteria. The sequences of the prophage regions from strains 12605 and 3663 were found to be homologous to those of *Vibrio* prophages. Integrative and conjugative elements (ICEs) are a diverse group of mobile elements found in bacteria^18^ that enable horizontal gene transfer (HGT) of virulence genes, antibiotic resistance, etc^19^. According to the ICEberg database, there are two well-characterized ICEs found in *V*. *fluvialis*, ICE*Vfl*H-08942 in *V*. *fluvialis* H-08942 (ICEberg ID 149) and ICE*Vfl*Ind1 in *V*. *fluvialis* Ind1(ICEberg ID 36). ICE*Vfl*Ind1was first discovered in *V*. *fluvialis* Ind1with resistance profile (*dfr18*, *floR*, *strBA* and *sul2*) and notable variable genes (toxin-antitoxin system^33^). A number of genes belonging to ICE*Vfl*Ind1 (ICEberg ID 36) were found in the nine *V*. *fluvialis* strains. Interestingly, strains 539 and 560 carry more ICE genes when compared to other strains. *V*. *fluvialis* strains 539 and 560, which inhabit *Crassostrea rhizophorae* and *Anomalocardia brasiliana* respectively, were also reported to possess a variant of the ICE SXT elements for the first time in Brazil^10^. SXT is a member of the ICE SXT/R391 family and was first described in clinical isolates of *V*. *cholerae* O139^34^.

Comparative analysis of the nine available genomes of *V*. *fluvialis* allowed us to determine the global gene repertoire of the species. The genome comparisons also revealed a ‘pan-genome’ that includes a core genome consisting of 3,147 genes common to all strains. This core-genome represents 71.6% of the genome of strain 12605 and 70.9% of the genome of strain 3663. Phylogenetic analysis divided the dataset into distinct populations, with the bile-isolated strains 12605 and 3663 being grouped into the same clade. Two animal-associated strains (539 and 560) were clustered together, showing a distinct separation from strains ATCC 33809 and NBRC 103150 isolated from human fecal samples. Strains PG41, I21563 and S1110 formed separate lineages.

Our study describes two cases of acute cholecystitis caused by *V*. *fluvialis* and provides new insights into the genomic architecture of the pathogen. The genomic information obtained in this study not only increases our understanding of the genetic basis of bile resistance, virulence, and adaptation mechanisms in *V*. *fluvialis*, but also helps in the identification of *V*. *fluvialis* core genes that can facilitate the detection of *V*. *fluvialis* in clinical samples.

## Methods

### Ethics approval statement

The study was conducted in accordance with the Declaration of Helsinki and was approved by the Clinical Ethics Committee of the first Affiliated Hospital of Zhejiang University. Written informed consent of this case report was obtained from the patient for the publication

### Identification of isolates

Both isolates were isolated from Mueller-Hinton broth agar with 5% sheep blood. The initial identification of strains 3663 and 12605 was done by MALDI-TOF MS analysis as previously described^35^. Overnight grown cultures were subjected to analysis using a Microflex MALDI-TOF mass spectrometer (BrukerDaltonics, Germany). The raw spectra were then analyzed using the MALDI Biotyper 2.0 database (BrukerDaltonics, Germany). The 16S rRNA nucleotide sequences of the two strains were obtained by PCR and sequencing. The sequences were then compared against NCBI database and EzTaxon-e database.

### Growth conditions and bile treatment

*V*. *fluvialis* strains were grown anaerobically at 37 °C in FEM medium (10 g of Bacto-Peptone (Difco) and 40 g of NaCl adjusted to pH 8.5, per liter) with or without 0.3% ox bile solution treatment; three biological replicates were performed.

### Antimicrobial susceptibility testing

Testing for susceptibility to amikacin, aztreonam, ciprofloxacin, meropenem, piperacillin, gentamicin, levofloxacin, cefepime, amoxicillin/clavulanic acid, imipenem, cefotaxime, ceftazidime, chloramphenicol, cefoperazone and minocycline was done by the disc diffusion method and interpreted with reference to a previous study^9^. *Escherichia coli* ATCC 25922 was used as control.

### Sequencing, assembly and annotation

Whole genome sequencing of strain 12605 was carried out using PacBio RS II Sequencing System and genome assembly was done by SMRT Analysis 2.2.1 ^36^. Whole genome sequencing of strain 3663 was carried out using the Illumina Hiseq. 2000 sequencer (Illumina, USA) with a high-throughput 2 × 100 bp pair end sequencing strategy. Prior to analysis, read sets were filtered, which involved deleting reads with low-quality base calls or similarity to Illumina adaptors. Subsequently, the raw reads were trimmed and assembled using Velvet. PAGIT flow was used to assemble the contigs and correct sequencing errors as described previously^37^. The two genomes were finally annotated by RAST server^38^.

### Identification of Integrative and Conjugative Elements (ICEs) and virulence-associated genes

Integrative and conjugative elements (ICEs) were identified using BLAST-2.3. 0+ program^20^ against the ICEberg database^19^ with an e-value cutoff of 1e-10 and an identity threshold of 60%. Virulence factors were annotated using BLAST-2.3. 0+ program^20^ against the virulence factor database (VFDB)^39^ with an e-value cutoff of 1e-10 and an identity threshold of 80%. Putative phage sequences were identified by PHAST^40^.

### Comparative genomic analysis

ANI values among *V*. *fluvilas* strains were calculated using OrthoANI^41^. Genomic BLAST file of *V*. *fluvilas* strains was downloaded from NCBI (https://www.ncbi.nlm.nih.gov/genome/2299) and the dendrogram was visualized using FigTree v.1.4 ^42^. Multiple genome alignment was performed using Mauve^43^ and BLAST Ring Image Generator (BRIG)^44^. For the pan-genome computation, PGAP v1.12 was used as described earlier^45^. The function of ortholog clusters was classified according to clusters of orthologous groups (COGs)^46^.

### Accession numbers

The 16S rRNA sequences of *V*. *fluvialis* 12605 and 3663 have been deposited in GenBank with the accession numbers KP780091 and KP780090. The complete genome sequence of *V*. *fluvialis* 12605 has been deposited in DDBJ/EMBL/GenBank with the accession numbers CP019118 and CP019119. Whole Genome Shotgun data of *V*. *fluvialis* 3663 has been deposited in DDBJ/EMBL/GenBank with the accession number JXXQ00000000.

## Electronic supplementary material

Supplementary Information

## References

[1] Ramamurthy T, Chowdhury G, Pazhani GP, Shinoda S (2014). Vibrio fluvialis: an emerging human pathogen. Front Microbiol.

[2] Huang KC, Hsu RW (2005). Vibrio fluvialis hemorrhagic cellulitis and cerebritis. Clinical infectious diseases: an official publication of the Infectious Diseases Society of America.

[3] Albert MJ (1991). A fatal case associated with shigellosis and Vibrio fluvialis bacteremia. Diagnostic microbiology and infectious disease.

[4] Lee JY (2008). Acute infectious peritonitis caused by Vibrio fluvialis. Diagnostic microbiology and infectious disease.

[5] Chen PJ, Tseng CC, Chan HT, Chao CM (2012). Acute Otitis due to Vibrio fluvialis after Swimming. Case reports in emergency medicine.

[6] Hassan IJ, MacGowan AP, Cook SD (1992). Endophthalmitis at the Bristol Eye Hospital: an 11-year review of 47 patients. The Journal of hospital infection.

[7] Yoshii Y, Nishino H, Satake K, Umeyama K (1987). Isolation of Vibrio fluvialis, and unusual pathogen in acute suppurative cholangitis. The American journal of gastroenterology.

[8] Liu WL, Chiu YH, Chao CM, Hou CC, Lai CC (2011). Biliary tract infection caused by Vibrio fluvialis in an immunocompromised patient. Infection.

[9] Liang P, Cui X, Du X, Kan B, Liang W (2013). The virulence phenotypes and molecular epidemiological characteristics of Vibrio fluvialis in China. Gut pathogens.

[10] de Oliveira Veras, A. A. *et al*. Draft Genome Sequences of Vibrio fluvialis Strains 560 and 539, Isolated from Environmental Samples. *Genome announcements* **3**, doi:10.1128/genomeA.01344-14 (2015). 10.1128/genomeA.01344-14 PMC4290981 25573928

[11] Khatri I, Mahajan S, Dureja C, Subramanian S, Raychaudhuri S (2013). Evidence of a new metabolic capacity in an emerging diarrheal pathogen: lessons from the draft genomes of Vibrio fluvialis strains PG41 and I21563. Gut pathogens.

[12] Yoon, S.-H., *et al*. Introducing EzBioCloud: A taxonomically united database of 16S rRNA and whole genome assemblies. *International journal of systematic and evolutionary microbiology* (2016). 10.1099/ijsem.0.001755 PMC5563544 28005526

[13] Goris J (2007). DNA–DNA hybridization values and their relationship to whole-genome sequence similarities. International journal of systematic and evolutionary microbiology.

[14] Richter M, Rosselló-Móra R (2009). Shifting the genomic gold standard for the prokaryotic species definition. Proceedings of the National Academy of Sciences.

[15] Chun J, Rainey FA (2014). Integrating genomics into the taxonomy and systematics of the Bacteria and Archaea. International journal of systematic and evolutionary microbiology.

[16] Zhang Z, Schwartz S, Wagner L, Miller W (2000). A greedy algorithm for aligning DNA sequences. Journal of Computational biology.

[17] Morgulis A (2008). Database indexing for production MegaBLAST searches. Bioinformatics.

[18] Burrus V, Pavlovic G, Decaris B, Guédon G (2002). Conjugative transposons: the tip of the iceberg. Molecular microbiology.

[19] Bi D (2012). ICEberg: a web-based resource for integrative and conjugative elements found in Bacteria. Nucleic acids research.

[20] Camacho C (2009). BLAST+: architecture and applications. BMC bioinformatics.

[21] Schauer K (2010). The Helicobacter pylori GroES cochaperonin HspA functions as a specialized nickel chaperone and sequestration protein through its unique C-terminal extension. Journal of bacteriology.

[22] Lu X (2014). Identification of genetic bases of vibrio fluvialis species-specific biochemical pathways and potential virulence factors by comparative genomic analysis. Appl Environ Microbiol.

[23] Casjens S (2003). Prophages and bacterial genomics: what have we learned so far?. Molecular microbiology.

[24] Merritt ME, Donaldson JR (2009). Effect of bile salts on the DNA and membrane integrity of enteric bacteria. Journal of medical microbiology.

[25] Hay AJ, Zhu J (2015). Host intestinal signal-promoted biofilm dispersal induces Vibrio cholerae colonization. Infection and immunity.

[26] Provenzano D, Schuhmacher DA, Barker JL, Klose KE (2000). The Virulence Regulatory Protein ToxR Mediates Enhanced Bile Resistance in Vibrio cholerae and Other PathogenicVibrio Species. Infection and immunity.

[27] Zhang X (2013). Functional genomic analysis of bile salt resistance in Enterococcus faecium. BMC genomics.

[28] Begley M, Gahan CG, Hill C (2005). The interaction between bacteria and bile. FEMS microbiology reviews.

[29] Bina JE, Mekalanos JJ (2001). Vibrio cholerae tolC is required for bile resistance and colonization. Infection and immunity.

[30] Lin J, Sahin O, Michel LO, Zhang Q (2003). Critical role of multidrug efflux pump CmeABC in bile resistance and *in vivo* colonization of Campylobacter jejuni. Infection and immunity.

[31] Hernández SB, Cota I, Ducret A, Aussel L, Casadesús J (2012). Adaptation and preadaptation of Salmonella enterica to bile. PLoS Genet.

[32] López-Garrido J, Cheng N, García-Quintanilla F (2010). García-del Portillo, F. & Casadesús, J. Identification of the Salmonella enterica damX gene product, an inner membrane protein involved in bile resistance. Journal of bacteriology.

[33] Wozniak RA (2009). Comparative ICE genomics: insights into the evolution of the SXT/R391 family of ICEs. PLoS genetics.

[34] Daccord A, Ceccarelli D, Burrus V (2010). Integrating conjugative elements of the SXT/R391 family trigger the excision and drive the mobilization of a new class of Vibrio genomic islands. Molecular microbiology.

[35] Espinal P, Seifert H, Dijkshoorn L, Vila J, Roca I (2012). Rapid and accurate identification of genomic species from the Acinetobacter baumannii (Ab) group by MALDI-TOF MS. Clinical microbiology and infection: the official publication of the European Society of Clinical Microbiology and Infectious Diseases.

[36] Liao Y-C, Lin S-H (2015). Lin, H.-H. Completing bacterial genome assemblies: strategy and performance comparisons. Scientific reports.

[37] Zhang F (2014). Permanent draft genome sequence of Bacillus flexus strain T6186-2, a multidrug-resistant bacterium isolated from a deep-subsurface oil reservoir. Marine genomics.

[38] Aziz RK (2008). The RAST Server: rapid annotations using subsystems technology. BMC genomics.

[39] Chen L, Xiong Z, Sun L, Yang J (2012). & Jin, Q. VFDB 2012 update: toward the genetic diversity and molecular evolution of bacterial virulence factors. Nucleic acids research.

[40] Zhou Y, Liang Y, Lynch KH, Dennis JJ, Wishart DS (2011). PHAST: a fast phage search tool. Nucleic acids research.

[41] Lee I, Kim YO, Park S-C, Chun J (2016). OrthoANI: an improved algorithm and software for calculating average nucleotide identity. International journal of systematic and evolutionary microbiology.

[42] Rambaut, A. 2006-2012. Fig. Tree. Tree Figure Drawing Tool, version 1.4. 0. *University of Edinburgh: Institute of Evolutionary Biology [on-line]*. Available from tree. bio. ed. ac. uk/ (accessed 30 January 2015).

[43] Darling AE, Mau B, Perna N (2010). T. progressiveMauve: multiple genome alignment with gene gain, loss and rearrangement. PloS one.

[44] Alikhan NF, Petty NK, Ben Zakour NL, Beatson SA (2011). BLAST Ring Image Generator (BRIG): simple prokaryote genome comparisons. BMC Genomics.

[45] Kim J-N (2015). Comparative genomics reveals the core and accessory genomes of Streptomyces species. J. Microbiol. Biotechnol.

[46] Tatusov RL (2001). The COG database: new developments in phylogenetic classification of proteins from complete genomes. Nucleic Acids Res.

