# Supplementary material for: Genome characterization of two bile-isolated Vibrio fluvialis strains: an insight into pathogenicity and bile salt adaption
Source: Sci Rep. 2017 Sep 19;7:11827. doi: 10.1038/s41598-017-12304-8 (PMC5605694; doi:10.1038/s41598-017-12304-8)
Supplement: Supplementary file 1 — Supplementary Information [file 41598_2017_12304_MOESM1_ESM.pdf]

Supplementary Information

Genome characterization of two bile-isolated *Vibrio fluvialis* strains: an insight into pathogenicity and bile salt adaption

Beiwen Zheng[1,#], Xiawei Jiang[2,#], Hong Cheng[3,#], Lihua Guo[1], Jing Zhang[1,4], Hao Xu[1], Xiao Yu[1], Chen Huang[1], Jinru Ji[1], Chaoqun Ying[1], Youjun Feng[5], Yonghong Xiao[1]* and Lanjuan Li[1]*

[1]State Key Laboratory for Diagnosis and Treatment of Infectious Disease, Collaborative Innovation Center for Diagnosis and Treatment of Infectious Diseases, The First Affiliated Hospital, College of Medicine, Zhejiang University, Hangzhou, China

[2]College of Basic Medical Sciences, Zhejiang Chinese Medical University, Hangzhou, China

[3]Key Laboratory of Marine Ecosystem and Biogeochemistry, Second Institute of Oceanography, State Oceanic Administration, Hangzhou, China

[4]Department of Respiratory Diseases, The First Affiliated Hospital, College of Medicine, Zhejiang University, Hangzhou, China

[5]Department of Medical Microbiology and Parasitology, Zhejiang University School of Medicine, Hangzhou, China

*Correspondence and requests for materials should be addressed to Y.X. (email: xiao-yonghong@163.com) or L.L. (email: ljli@zju.edu.cn)

Table S1. Antimicrobial susceptibility of *V. fluvialis* strain 12605 and 3663 to different antibiotics according to CLSI guidelines

Antibiotic	12605		3663	
	Disc diffusion (mm)	Susceptibility	Disc diffusion (mm)	Susceptibility
Amikacin	19	Susceptible	20	Susceptible
Aztreonam	24	Susceptible	23	Susceptible
Ciprofloxacin	23	Susceptible	23	Susceptible
Meropenem	23	Susceptible	23	Susceptible
Piperacillin	23	Susceptible	22	Susceptible
Piperacillin/ Tazobactam	23	Susceptible	21	Susceptible
Gentamycin	19	Susceptible	18	Susceptible
Levofloxacin	25	Susceptible	25	Susceptible
Cefepime	28	Susceptible	25	Susceptible
Cefoperazone/ Sulbactam	28	Susceptible	25	Susceptible
Imipenem	23	Susceptible	21	Susceptible
Cefotaxime	28	Susceptible	31	Susceptible
Ceftazidime	27	Susceptible	26	Susceptible
Chloramphenicol	28	Susceptible	29	Susceptible
Ticarcillin/ Clavulanic acid	22	Susceptible	22	Susceptible
Cefoperazone	27	Susceptible	25	Susceptible
Minocycline	24	Susceptible	22	Susceptible

Table S2. Integrative and Conjugative Elements identified in *V. fluvialis* 12605

Sequence ID	ICEbergID	identity (%)	alignment length
fig|6666666.230735.peg.444	ICEberg|36gi|259156615|gb|ACV96558.1|ribosomal protein S15[Vibrio fluvialis Ind1]	95.506	89
fig|6666666.230735.peg.3608	ICEberg|360gi|29140228|gb|AAO71792.1|phage integrase[Salmonella entericasub sp. entericaserovar Typhistr.Ty2]	94.118	34
fig|6666666.230735.peg.457	ICEberg|16gi|229371393|gb|ACQ61816.1|peptide chain release factor 3[Vibrio cholerae MJ-1236]	91.871	529
fig|6666666.230735.peg.450	ICEberg|36gi|259156579|gb|ACV96522.1|STM-proteaseA[Vibrio fluvialis Ind1]	91.159	328
fig|6666666.230735.peg.445	ICEberg|36gi|259156595|gb|ACV96538.1|polyribonucleotide nucleotidyltransferase[Vibrio fluvialis Ind1]	90.858	711
fig|6666666.230735.peg.462	ICEberg|36gi|259156631|gb|ACV96574.1|lysyl-tRNA synthetase(Lysine--tRNAligase)(LysRS)[Vibrio fluvialis Ind1]	88.85	287
fig|6666666.230735.peg.458	ICEberg|36gi|259156570|gb|ACV96513.1|ATP-dependent RNA helicase SrmB[Vibrio fluvialis Ind1]	88.654	379
fig|6666666.230735.peg.449	ICEberg|36gi|259156589|gb|ACV96532.1|peptidase,U32family[Vibrio fluvialis Ind1]	86.598	291
fig|6666666.230735.peg.443	ICEberg|36gi|259156613|gb|ACV96556.1|tRNA pseudouridine synthase B[Vibrio fluvialis Ind1]	83.226	310
fig|6666666.230735.peg.452	ICEberg|36gi|259156584|gb|ACV96527.1|sterol binding protein[Vibrio fluvialis Ind1]	83.108	148
fig|6666666.230735.peg.3089	ICEberg|23gi|259156500|gb|ACV96444.1|filamentation induced by cAMP protein Fic[Vibrio cholerae Mex1]	80.22	364
fig|6666666.230735.peg.453	ICEberg|36gi|259156550|gb|ACV96493.1|acetyltransferase,gnat family[Vibrio fluvialis Ind1]	79.641	167
fig|6666666.230735.peg.448	ICEberg|36gi|259156626|gb|ACV96569.1|multidrug resistance pump[Vibrio fluvialis Ind1]	76.802	444
fig|6666666.230735.peg.1467	ICEberg|381gi|306529204|gb|ADM99134.1|Hcp[Dickeya dadantii 3937]	76.744	172
fig|6666666.230735.peg.3245	ICEberg|381gi|306529204|gb|ADM99134.1|Hcp[Dickeya dadantii 3937]	76.744	172
fig|6666666.230735.peg.3888	ICEberg|381gi|306529204|gb|ADM99134.1|Hcp[Dickeya dadantii 3937]	76.744	172

fig|6666666.230735.peg.460	ICEberg|36gi|259156552|gb|ACV96495.1|branched-chain amino acid transport system II carrier protein[Vibrio fluvialis Ind1]	74.828	437
fig|6666666.230735.peg.456	ICEberg|36gi|259156587|gb|ACV96530.1|ggdef domain protein[Vibrio fluvialis Ind1]	74.521	679
fig|6666666.230735.peg.2085	ICEberg|53gi|133739134|emb|CAL62183.1|Alcohol dehydrogenase class-3(Alcohol dehydrogenase class-III) (S-(hydroxymethyl)glutathione dehydrogenase)(Glutathione-dependent formaldehyde dehydrogenase)(FDH)(FALDH)[Herminiimon asarsenicoxydans]	73.442	369
fig|6666666.230735.peg.442	ICEberg|36gi|259156566|gb|ACV96509.1|ribosome-binding factor A[Vibrio fluvialis Ind1]	72.093	43
fig|6666666.230735.peg.3980	ICEberg|287gi|238815137|gb|ACR56726.1|pentapeptide repeat[Escherichia coli]	71.56	218
fig|6666666.230735.peg.455	ICEberg|36gi|259156594|gb|ACV96537.1|ribosomal-protein-alanineacetyl transferase[Vibrio fluvialis Ind1]	71.141	149
fig|6666666.230735.peg.2132	ICEberg|52gi|218771568|emb|CAW27339.1|[2Fe-2S]ferredoxin[Pseudomonas aeruginosa LESB58]	70.536	112
fig|6666666.230735.peg.1214	ICEberg|113gi|327412869|emb|CAX67877.1|putative exported sulfatase family protein[Salmonella bongori]	70.488	471
fig|6666666.230735.peg.447	ICEberg|36gi|259156612|gb|ACV96555.1|transcriptiona lregulator,MarR family[Vibrio fluvialis Ind1]	70.068	147
fig|6666666.230735.peg.1653	ICEberg|50gi|78036297|emb|CAJ23988.1|thioredoxin reductase[Xanthomonas campestris pv.vesicatoria str.85-10]	69.427	314
fig|6666666.230735.peg.2074	ICEberg|71gi|297592322|gb|ADI47060.1|putative ribosomal protein[Escherichia coli]	69.412	85
fig|6666666.230735.peg.1860	ICEberg|338gi|93354309|gb|ABF08398.1|glyceraldehyde-3-phosphate dehydrogenase A(GAPDH-A)[Cupriavidus metallidurans CH34]	69.207	328
fig|6666666.230735.peg.2030	ICEberg|391gi|149932528|gb|ABR39226.1|putative dehydratase[Bacteroides vulgatus ATCC8482]	69.027	339
fig|6666666.230735.peg.123	ICEberg|133gi|14025593|dbj|BAB52193.1|chaperonin GroEL[Mesorhizobium loti MAFF303099]	68.998	529

fig|6666666.230735.peg.3319	ICEberg|204gi|190011241|emb|CAQ44853.1|putative 4-carboxymuconolactone decarboxylase[Stenotrophomonas maltophilia K279a]	68.852	122
fig|6666666.230735.peg.388	ICEberg|76gi|157419732|gb|ABV55421.1|ABC transporter ATP-subunit YadG-like protein[Streptococcus dysgalactiae subsp.equisimilis]	68.092	304
fig|6666666.230735.peg.3619	ICEberg|394gi|281376355|gb|ADA65845.1|cold-shock protein[Lactococcus lactissub sp.lactis KF147]	67.742	62
fig|6666666.230735.peg.1215	ICEberg|113gi|327412870|emb|CAX67878.1|putative arylsulfatase-activating protein[Salmonella bongori]	67.303	419
fig|6666666.230735.peg.2729	ICEberg|75gi|20804219|emb|CAD31245.1|PROBABLE THIAMIN BIOSYNTHESIS PROTEIN THIC[Mesorhizobium loti R7A]	66.889	601
fig|6666666.230735.peg.389	ICEberg|76gi|157419731|gb|ABV55420.1|inner membrane transport permease YadH-like protein[Streptococcus dysgalactiaesubsp.equisimilis]	66.797	256
fig|6666666.230735.peg.270	ICEberg|54gi|237501459|gb|ACQ94052.1|ornithine carbamoyltransferase[Tolumonas auensis DSM 9187]	66.766	334
fig|6666666.230735.peg.3553	ICEberg|75gi|20804075|emb|CAD31278.1|HYPOTHETICAL 2-AMINO-3-KETOBUTYRATE COENZYME A LIGASE 2.3.1.29 PROTEIN[Mesorhizobium loti R7A]	66.751	397
fig|6666666.230735.peg.160	ICEberg|338gi|93354304|gb|ABF08393.1|D-ribulose-5-phosphate3-epimerase[Cupriavidus metallidurans CH34]	66.364	220
fig|6666666.230735.peg.3897	ICEberg|235gi|68346359|gb|AAY93965.1|cytochrome o ubiquino loxidase,subunit I [Pseudomonas fluorescens Pf-5]	65.991	641
fig|6666666.230735.peg.1503	ICEberg|75gi|20804070|emb|CAD31273.1|PUTATIVE AMINO-ACID ABC TRANSPORTER ATP-BINDING PROTEIN[Mesorhizobium loti R7A]	65.447	246
fig|6666666.230735.peg.81	ICEberg|54gi|237501459|gb|ACQ94052.1|ornithine carbamoyltransferase[Tolumonas auensis DSM 9187]	65.373	335
fig|6666666.230735.peg.4280	ICEberg|75gi|20804205|emb|CAD31231.1|PROBABLE CHAPERONIN GROEL DF PROTEIN[Mesorhizobium lotiR 7A]	65.348	531

fig|6666666.230735.peg.2446	ICEberg|360gi|29140135|gb|AAO71699.1|single strand binding protein[Salmonella entericasub sp. entericaserovar Typhistr.Ty2]	64.773	176
fig|6666666.230735.peg.2117	ICEberg|125gi|13274515|gb|AAK17959.1|AF329848_11GMP459[Clostridium perfringens]	63.83	47
fig|6666666.230735.peg.3432	ICEberg|192gi|187726600|gb|ACD27765.1|Electron transfer flavoprotein alpha/beta-subunit[Ralstonia pickettii 12J]	63.454	249
fig|6666666.230735.peg.642	ICEberg|52gi|218771593|emb|CAW27366.1|2-dehydro-3-deoxyphosphooctonate aldolase[Pseudomonas aeruginosa LESB58]	63.235	272
fig|6666666.230735.peg.1529	ICEberg|173gi|37955684|gb|AAP22523.1|CspA[Pseudomonas aeruginosa]	63.235	68
fig|6666666.230735.peg.2770	ICEberg|128gi|270344715|gb|ACZ77480.1|twin-arginine translocation protein,TatA/E family subunit[Dickeya dadantii Ech586]	62.963	54
fig|6666666.230735.peg.2373	ICEberg|75gi|20803994|emb|CAD31571.1|PROBABLE S-ADENOSYLMETHIONINE SYNTHETAS EPROTEIN[Mesorhizobium loti R7A]	62.76	384
fig|6666666.230735.peg.1414	ICEberg|133gi|14026116|dbj|BAB52714.1|cytochrome-c oxidase FixN chain[Mesorhizobium loti MAFF303099]	62.69	461
fig|6666666.230735.peg.1210	ICEberg|394gi|281376355|gb|ADA65845.1|cold-shock protein[Lactococcus lactissub sp.lactis KF147]	62.5	64
fig|6666666.230735.peg.1296	ICEberg|133gi|14025844|dbj|BAB52443.1|fumarate hydratase,classI[Mesorhizobium loti MAFF303099]	61.6	500
fig|6666666.230735.peg.1085	ICEberg|133gi|14025612|dbj|BAB52212.1|biotin synthetase[Mesorhizobium loti MAFF303099]	61.218	312
fig|6666666.230735.peg.934	ICEberg|394gi|281376355|gb|ADA65845.1|cold-shock protein[Lactococcus lactissub sp.lactis KF147]	60.938	64
fig|6666666.230735.peg.948	ICEberg|218gi|310759034|gb|ADP14483.1|cation efflux system protein CusA[Achromobacte rxylosoxidans A8]	60.653	1042

fig|6666666.230735.peg.3896	ICEberg|235gi|68346358|gb|AAY93964.1|cytochrome o ubiquinol oxidase,subunit III[Pseudomonas fluorescens Pf-5]	60.606	198
fig|6666666.230735.peg.2086	ICEberg|53gi|133739131|emb|CAL62180.1|S-formylglutathione hydrolase[Herminiimonas arsenicoxydans]	60.595	269
fig|6666666.230735.peg.3232	ICEberg|202gi|221732141|gb|ACM34961.1|DNA repair protein RadC[Acidovorax ebreus TPSY]	60.563	142
fig|6666666.230735.peg.3702	ICEberg|242gi|32261794|gb|AAP76844.1|conserved hypothetical protein[Helicobacter hepaticus ATCC51449]	60.46	478
fig|6666666.230735.peg.3727	ICEberg|195gi|120587861|gb|ABM31301.1|major facilitator superfamily MFS_1[Acidovorax citrulli AAC00-1]	60.363	386
fig|6666666.230735.peg.86	ICEberg|54gi|237501461|gb|ACQ94054.1|C4-dicarboxylate anaerobic carrier[Tolumonas auensis DSM9187]	60.345	464
fig|6666666.230735.peg.3468	ICEberg|133gi|14025769|dbj|BAB52368.1|acetyl/propionyl CoA carboxylase,beta subunit[Mesorhizobium loti MAFF303099]	60	535

Table S3. Integrative and Conjugative Elements identified in *V. fluvialis* 3663

Sequence ID	ICEbergID	identity (%)	alignment length
fig|6666666.230986.peg.2458	ICEberg|76gi|157419732|gb|ABV55421.1|ABC transporter ATP-subunit YadG-like protein[Streptococcus dysgalactiae subsp.equisimilis]	68.092	304
fig|6666666.230986.peg.2457	ICEberg|76gi|157419731|gb|ABV55420.1|inner membrane transport permease YadH-like protein[Streptococcus dysgalactiaesubsp.equisimilis]	66.797	256
fig|6666666.230986.peg.1295	ICEberg|75gi|20804205|emb|CAD31231.1|PROBABLE CHAPERONIN GROEL DF PROTEIN[Mesorhizobium lotiR 7A]	65.725	531
fig|6666666.230986.peg.3052	ICEberg|75gi|20804075|emb|CAD31278.1|HYPOTHETICAL 2-AMINO-3-KETOBUTYRATE COENZYME A LIGASE 2.3.1.29 PROTEIN[Mesorhizobium loti R7A]	66.499	397
fig|6666666.230986.peg.2982	ICEberg|75gi|20804070|emb|CAD31273.1|PUTATIVE AMINO-ACID ABC TRANSPORTER ATP-BINDING PROTEIN[Mesorhizobium loti R7A]	65.447	246
fig|6666666.230986.peg.782	ICEberg|75gi|20803994|emb|CAD31571.1|PROBABLE S-ADENOSYLMETHIONINE SYNTHETAS EPROTEIN[Mesorhizobium loti R7A]	62.76	384
fig|6666666.230986.peg.93	ICEberg|71gi|297592322|gb|ADI47060.1|putative ribosomal protein[Escherichia coli]	72.5	80
fig|6666666.230986.peg.3111	ICEberg|54gi|237501461|gb|ACQ94054.1|C4-dicarboxylate anaerobic carrier[Tolumonas auensis DSM9187]	60.129	464
fig|6666666.230986.peg.4108	ICEberg|54gi|237501459|gb|ACQ94052.1|ornithine carbamoyltransferase[Tolumonas auensis DSM 9187]	67.066	334
fig|6666666.230986.peg.3115	ICEberg|54gi|237501459|gb|ACQ94052.1|ornithine carbamoyltransferase[Tolumonas auensis DSM 9187]	65.373	335
fig|6666666.230986.peg.104	ICEberg|53gi|133739134|emb|CAL62183.1|Alcohol dehydrogenase class-3(Alcohol dehydrogenase class-III)(S-(hydroxymethyl)glutathione dehydrogenase)(Glutathione-dependent formaldehyde dehydrogenase)(FDH)(FALDH)[Herminiimon asarsenicoxydans]	73.442	369

fig|6666666.230986.peg.105	ICEberg|53gi|133739131|emb|CAL62180.1|S-formylglutathione hydrolase[Herminiimonas arsenicoxydans]	60.223	269
fig|6666666.230986.peg.1360	ICEberg|52gi|218771593|emb|CAW27366.1|2-dehydro-3-deoxyphosphooctonate aldolase[Pseudomonas aeruginosa LESB58]	63.235	272
fig|6666666.230986.peg.1240	ICEberg|52gi|218771568|emb|CAW27339.1|[2Fe-2S]ferredoxin[Pseudomonas aeruginosa LESB58]	70.536	112
fig|6666666.230986.peg.3512	ICEberg|50gi|78036297|emb|CAJ23988.1|thioredoxin reductase[Xanthomonas campestris pv.vesicatoria str.85-10]	69.427	314
fig|6666666.230986.peg.3944	ICEberg|43gi|20095169|gb|AAM08035.1|putative transposase tnp391A[Providencia rettgeri]	62.559	211
fig|6666666.230986.peg.111	ICEberg|434gi|14972539|gb|AAK75178.1|transposase,IS200 family[Streptococcus pneumoniae TIGR4]	61.616	99
fig|6666666.230986.peg.3194	ICEberg|39gi|120558048|gb|ABM23975.1|transposase IS3/IS911 family protein[Shewanella sp.W3-18-1]	61.818	55
fig|6666666.230986.peg.3193	ICEberg|39gi|120558047|gb|ABM23974.1|Integrase,catalytic region[Shewanella sp.W3-18-1]	65.724	283
fig|6666666.230986.peg.1727	ICEberg|394gi|281376355|gb|ADA65845.1|cold-shock protein[Lactococcus lactissub sp.lactis KF147]	67.742	62
fig|6666666.230986.peg.4268	ICEberg|394gi|281376355|gb|ADA65845.1|cold-shock protein[Lactococcus lactissub sp.lactis KF147]	62.5	64
fig|6666666.230986.peg.3597	ICEberg|394gi|281376355|gb|ADA65845.1|cold-shock protein[Lactococcus lactissub sp.lactis KF147]	60.938	64
fig|6666666.230986.peg.1224	ICEberg|384gi|115587014|gb|ABJ13029.1|putative integrase[Pseudomonas aeruginosaUCBPP-PA14]	86.802	394
fig|6666666.230986.peg.1217	ICEberg|384gi|115587001|gb|ABJ13016.1|mating pair formation protein TrbJ[Pseudomonas aeruginosaUCBPP-PA14]	69.456	239

fig|6666666.230986.peg.821	ICEberg|381gi|306529204|gb|ADM99134.1|Hcp[Dickeya dadantii 3937]	76.866	134
fig|6666666.230986.peg.78	ICEberg|381gi|306529204|gb|ADM99134.1|Hcp[Dickeya dadantii 3937]	76.866	134
fig|6666666.230986.peg.2321	ICEberg|381gi|306529204|gb|ADM99134.1|Hcp[Dickeya dadantii 3937]	76.163	172
fig|6666666.230986.peg.2652	ICEberg|36gi|259156631|gb|ACV96574.1|lysyl-tRNA synthetase(Lysine--tRNAligase)(LysRS)[Vibrio fluvialis Ind1]	88.85	287
fig|6666666.230986.peg.2638	ICEberg|36gi|259156626|gb|ACV96569.1|multidrug resistance pump[Vibrio fluvialis Ind1]	76.802	444
fig|6666666.230986.peg.2634	ICEberg|36gi|259156615|gb|ACV96558.1|ribosomal protein S15[Vibrio fluvialis Ind1]	95.506	89
fig|6666666.230986.peg.2633	ICEberg|36gi|259156613|gb|ACV96556.1|tRNA pseudouridine synthase B[Vibrio fluvialis Ind1]	83.548	310
fig|6666666.230986.peg.2637	ICEberg|36gi|259156612|gb|ACV96555.1|transcriptiona lregulator,MarR family[Vibrio fluvialis Ind1]	72.388	134
fig|6666666.230986.peg.2635	ICEberg|36gi|259156595|gb|ACV96538.1|polyribonucleotide nucleotidyltransferase[Vibrio fluvialis Ind1]	90.858	711
fig|6666666.230986.peg.2645	ICEberg|36gi|259156594|gb|ACV96537.1|ribosomal-protein-alanineacetyl transferase[Vibrio fluvialis Ind1]	70.47	149
fig|6666666.230986.peg.2639	ICEberg|36gi|259156589|gb|ACV96532.1|peptidase,U32family[Vibrio fluvialis Ind1]	86.942	291
fig|6666666.230986.peg.2646	ICEberg|36gi|259156587|gb|ACV96530.1|ggdef domain protein[Vibrio fluvialis Ind1]	74.08	679
fig|6666666.230986.peg.2642	ICEberg|36gi|259156584|gb|ACV96527.1|sterol binding protein[Vibrio fluvialis Ind1]	83.108	148

fig|6666666.230986.peg.2640	ICEberg|36gi|259156579|gb|ACV96522.1|STM-proteaseA[Vibrio fluvialis Ind1]	91.159	328
fig|6666666.230986.peg.2648	ICEberg|36gi|259156570|gb|ACV96513.1|ATP-dependent RNA helicase SrmB[Vibrio fluvialis Ind1]	88.654	379
fig|6666666.230986.peg.2632	ICEberg|36gi|259156566|gb|ACV96509.1|ribosome-binding factor A[Vibrio fluvialis Ind1]	72.093	43
fig|6666666.230986.peg.2650	ICEberg|36gi|259156552|gb|ACV96495.1|branched-chain amino acid transport system II carrier protein[Vibrio fluvialis Ind1]	74.828	437
fig|6666666.230986.peg.2643	ICEberg|36gi|259156550|gb|ACV96493.1|acetyltransferase,gnat family[Vibrio fluvialis Ind1]	79.641	167
fig|6666666.230986.peg.1738	ICEberg|360gi|29140228|gb|AAO71792.1|phage integrase[Salmonella entericasub sp. entericaserovar Typhistr.Ty2]	94.118	34
fig|6666666.230986.peg.2532	ICEberg|360gi|29140135|gb|AAO71699.1|single strand binding protein[Salmonella entericasub sp. entericaserovar Typhistr.Ty2]	64.773	176
fig|6666666.230986.peg.846	ICEberg|338gi|93354309|gb|ABF08398.1|glyceraldehyde-3-phosphate dehydrogenase A(GAPDH-A)[Cupriavidus metallidurans CH34]	69.207	328
fig|6666666.230986.peg.3882	ICEberg|338gi|93354304|gb|ABF08393.1|D-ribulose-5-phosphate3-epimerase[Cupriavidus metallidurans CH34]	66.364	220
fig|6666666.230986.peg.1469	ICEberg|287gi|238815137|gb|ACR56726.1|pentapeptide repeat[Escherichia coli]	71.759	216
fig|6666666.230986.peg.3268	ICEberg|242gi|32261794|gb|AAP76844.1|conserved hypothetical protein[Helicobacter hepaticus ATCC51449]	60.46	478
fig|6666666.230986.peg.2102	ICEberg|23gi|259156500|gb|ACV96444.1|filamentation induced by cAMP protein Fic[Vibrio cholerae Mex1]	79.945	364
fig|6666666.230986.peg.1136	ICEberg|235gi|68346359|gb|AAY93965.1|cytochrome o ubiquino loxidase,subunit I [Pseudomonas fluorescens Pf-5]	65.991	641

fig|6666666.230986.peg.1137	ICEberg|235gi|68346358|gb|AAY93964.1|cytochrome o ubiquinol oxidase,subunit III[Pseudomonas fluorescens Pf-5]	60.606	198
fig|6666666.230986.peg.489	ICEberg|204gi|190011241|emb|CAQ44853.1|putative 4-carboxymuconolactone decarboxylase[Stenotrophomonas maltophilia K279a]	70.27	74
fig|6666666.230986.peg.490	ICEberg|204gi|190011241|emb|CAQ44853.1|putative 4-carboxymuconolactone decarboxylase[Stenotrophomonas maltophilia K279a]	63.043	46
fig|6666666.230986.peg.2334	ICEberg|202gi|221732141|gb|ACM34961.1|DNA repair protein RadC[Acidovorax ebreus TPSY]	60.563	142
fig|6666666.230986.peg.257	ICEberg|195gi|120587861|gb|ABM31301.1|major facilitator superfamily MFS_1[Acidovorax citrulli AAC00-1]	60.363	386
fig|6666666.230986.peg.4348	ICEberg|192gi|187726600|gb|ACD27765.1|Electron transfer flavoprotein alpha/beta-subunit[Ralstonia pickettii 12J]	63.855	249
fig|6666666.230986.peg.3197	ICEberg|176gi|148807278|gb|ABR13352.1|insertion sequence IS407 OrfB[Pseudomonas aeruginosa]	69.048	84
fig|6666666.230986.peg.2956	ICEberg|173gi|37955684|gb|AAP22523.1|CspA[Pseudomonas aeruginosa]	63.235	68
fig|6666666.230986.peg.2647	ICEberg|16gi|229371393|gb|ACQ61816.1|peptide chain release factor 3[Vibrio cholerae MJ-1236]	91.871	529
fig|6666666.230986.peg.3289	ICEberg|133gi|14026116|dbj|BAB52714.1|cytochrome-c oxidase FixN chain[Mesorhizobium loti MAFF303099]	62.69	461
fig|6666666.230986.peg.4181	ICEberg|133gi|14025844|dbj|BAB52443.1|fumarate hydratase,classI[Mesorhizobium loti MAFF303099]	61.6	500
fig|6666666.230986.peg.3922	ICEberg|133gi|14025769|dbj|BAB52368.1|acetyl/propionyl CoA carboxylase,beta subunit[Mesorhizobium loti MAFF303099]	60	535
fig|6666666.230986.peg.2727	ICEberg|133gi|14025612|dbj|BAB52212.1|biotin synthetase[Mesorhizobium loti MAFF303099]	61.218	312

fig|6666666.230986.peg.3847	ICEberg|133gi|14025593|dbj|BAB52193.1|chaperonin GroEL[Mesorhizobium loti MAFF303099]	68.998	529
fig|6666666.230986.peg.232	ICEberg|133gi|14025579|dbj|BAB52179.1|thiamin biosynthesis protein;ThiC[MesorhizobiumlotiMAFF303099]	66.113	602
fig|6666666.230986.peg.3196	ICEberg|129gi|218427568|emb|CAR08342.1|Transposase,ORFA,IS3family,IS407group[Escherichia coliED1a]	62.069	87
fig|6666666.230986.peg.2571	ICEberg|128gi|270344715|gb|ACZ77480.1|twin-arginine translocation protein,TatA/E family subunit[Dickeya dadantii Ech586]	62.963	54
fig|6666666.230986.peg.1225	ICEberg|125gi|13274515|gb|AAK17959.1|AF329848_11GMP459[Clostridium perfringens]	63.83	47
fig|6666666.230986.peg.4263	ICEberg|113gi|327412870|emb|CAX67878.1|putative arylsulfatase-activating protein[Salmonella bongori]	67.299	422
fig|6666666.230986.peg.4264	ICEberg|113gi|327412869|emb|CAX67877.1|putative exported sulfatase family protein[Salmonella bongori]	70.276	471

Table S4. Virulence factors identified in *V. fluvialis* 12605

Sequence ID	VFDB ID	identity (%)	alignment length
fig|6666666.230735.peg.3743	VFG044042(gi:23663958)	98.784	740
fig|6666666.230735.peg.751	VFG007568(gi:15642065)	96.825	126
fig|6666666.230735.peg.749	VFG007580(gi:15642067)	96.61	295
fig|6666666.230735.peg.620	VFG007352(gi:15642198)	96.377	138
fig|6666666.230735.peg.757	VFG007544(gi:15642059)	96.341	164
fig|6666666.230735.peg.2062	VFG007601(gi:27363787)	95.635	252
fig|6666666.230735.peg.1467	VFG007138(gi:15600788)	95.349	172
fig|6666666.230735.peg.3245	VFG007138(gi:15600788)	95.349	172
fig|6666666.230735.peg.3888	VFG007138(gi:15600788)	95.349	172
fig|6666666.230735.peg.618	VFG007364(gi:15642200)	93.818	275
fig|6666666.230735.peg.691	VFG007424(gi:15642124)	93.382	136
fig|6666666.230735.peg.684	VFG007466(gi:15642131)	93.003	343
fig|6666666.230735.peg.686	VFG007454(gi:15642129)	92.694	438
fig|6666666.230735.peg.3702	VFG002093(gi:15600879)	92.668	491
fig|6666666.230735.peg.877	VFG043132(gi:28900124)	92.337	261
fig|6666666.230735.peg.690	VFG007430(gi:15642125)	92.264	349
fig|6666666.230735.peg.624	VFG007328(gi:15642194)	91.985	262
fig|6666666.230735.peg.615	VFG007382(gi:15642203)	91.837	49
fig|6666666.230735.peg.619	VFG007358(gi:15642199)	91.603	131
fig|6666666.230735.peg.747	VFG007594(gi:28899009)	91.559	699
fig|6666666.230735.peg.613	VFG043121(gi:15642205)	91.538	130
fig|6666666.230735.peg.2306	VFG018241(gi:15640579)	91.279	172
fig|6666666.230735.peg.750	VFG007574(gi:15642066)	91.192	193
fig|6666666.230735.peg.621	VFG007346(gi:15642197)	91.064	235

fig|6666666.230735.peg.3696	VFG002082(gi:15600885)	90.541	444
fig|6666666.230735.peg.612	VFG043122(gi:15642206)	90.521	211
fig|6666666.230735.peg.617	VFG007370(gi:15642201)	90.26	308
fig|6666666.230735.peg.183	VFG007616(gi:15642596)	90.047	211
fig|6666666.230735.peg.626	VFG007316(gi:15642192)	90.028	361
fig|6666666.230735.peg.623	VFG007334(gi:15642195)	89.96	249
fig|6666666.230735.peg.902	VFG043156(gi:28901401)	89.813	697
fig|6666666.230735.peg.3701	VFG002094(gi:15600880)	89.655	145
fig|6666666.230735.peg.42	VFG007087(gi:27364322)	89.116	147
fig|6666666.230735.peg.679	VFG007496(gi:15642136)	89.035	456
fig|6666666.230735.peg.678	VFG007504(gi:28899028)	88.971	136
fig|6666666.230735.peg.2061	VFG007608(gi:37679056)	88.854	314
fig|6666666.230735.peg.915	VFG043143(gi:28901388)	88.789	446
fig|6666666.230735.peg.3703	VFG002092(gi:15600878)	88.485	165
fig|6666666.230735.peg.673	VFG007532(gi:15642142)	88.329	377
fig|6666666.230735.peg.2382	VFG042879(gi:27364897)	88.116	345
fig|6666666.230735.peg.696	VFG007394(gi:15642119)	87.87	338
fig|6666666.230735.peg.2578	VFG007659(gi:59710773)	87.749	351
fig|6666666.230735.peg.906	VFG043152(gi:28901397)	87.705	244
fig|6666666.230735.peg.694	VFG007409(gi:27365291)	87.64	89
fig|6666666.230735.peg.625	VFG007322(gi:15642193)	87.603	242
fig|6666666.230735.peg.622	VFG007340(gi:15642196)	87.558	434
fig|6666666.230735.peg.2559	VFG007651(gi:15640289)	87.477	551
fig|6666666.230735.peg.682	VFG007478(gi:15642133)	87.379	103
fig|6666666.230735.peg.910	VFG043148(gi:28901393)	87.133	443
fig|6666666.230735.peg.630	VFG007292(gi:15642187)	87.071	379

fig|6666666.230735.peg.873	VFG043128(gi:28900120)	86.806	144
fig|6666666.230735.peg.3694	VFG002084(gi:15600887)	86.782	870
fig|6666666.230735.peg.683	VFG007472(gi:15642132)	86.747	581
fig|6666666.230735.peg.2381	VFG042880(gi:27364898)	86.685	368
fig|6666666.230735.peg.2048	VFG045346(gi:37679074)	86.617	269
fig|6666666.230735.peg.372	VFG006908(gi:15642422)	86.52	408
fig|6666666.230735.peg.905	VFG043153(gi:28901398)	86.517	89
fig|6666666.230735.peg.2438	VFG006965(gi:27364824)	86.237	574
fig|6666666.230735.peg.875	VFG043130(gi:28900122)	86.181	398
fig|6666666.230735.peg.40	VFG007096(gi:15642726)	86.083	503
fig|6666666.230735.peg.3695	VFG002083(gi:15600886)	85.992	257
fig|6666666.230735.peg.680	VFG007490(gi:15642135)	85.714	350
fig|6666666.230735.peg.879	VFG043134(gi:28900126)	85.523	373
fig|6666666.230735.peg.41	VFG007090(gi:15642725)	85.504	407
fig|6666666.230735.peg.681	VFG007487(gi:27365278)	85.35	471
fig|6666666.230735.peg.693	VFG007412(gi:15642122)	85.294	272
fig|6666666.230735.peg.3690	VFG002088(gi:15600891)	85.267	1181
fig|6666666.230735.peg.3700	VFG002078(gi:15600881)	85.229	589
fig|6666666.230735.peg.876	VFG043131(gi:28900123)	85.185	243
fig|6666666.230735.peg.754	VFG007553(gi:27365301)	84.817	382
fig|6666666.230735.peg.3697	VFG002081(gi:15600884)	84.81	158
fig|6666666.230735.peg.1928	VFG007611(gi:147674163)	84.694	294
fig|6666666.230735.peg.631	VFG007286(gi:15642186)	84.615	377
fig|6666666.230735.peg.671	VFG007538(gi:15642143)	84.309	376
fig|6666666.230735.peg.674	VFG007528(gi:28899032)	84.309	376
fig|6666666.230735.peg.3498	VFG007245(gi:15600997)	84.244	311

fig|6666666.230735.peg.592	VFG013418(gi:16273103)	84.211	190
fig|6666666.230735.peg.898	VFG043159(gi:28901406)	83.594	128
fig|6666666.230735.peg.893	VFG043164(gi:28901411)	83.509	285
fig|6666666.230735.peg.872	VFG043127(gi:28900119)	83.333	120
fig|6666666.230735.peg.904	VFG043154(gi:28901399)	83.333	258
fig|6666666.230735.peg.39	VFG007106(gi:37678398)	83.013	677
fig|6666666.230735.peg.748	VFG007586(gi:15642068)	82.738	504
fig|6666666.230735.peg.695	VFG007404(gi:37680653)	82.692	260
fig|6666666.230735.peg.913	VFG043145(gi:28901390)	82.596	339
fig|6666666.230735.peg.912	VFG043146(gi:28901391)	82.534	584
fig|6666666.230735.peg.2577	VFG007661(gi:59710774)	82.517	286
fig|6666666.230735.peg.903	VFG043155(gi:28901400)	82.447	376
fig|6666666.230735.peg.4183	VFG044437(gi:42491184)	82.386	176
fig|6666666.230735.peg.373	VFG006902(gi:15642421)	82.028	562
fig|6666666.230735.peg.914	VFG043144(gi:28901389)	82.008	239
fig|6666666.230735.peg.3699	VFG007117(gi:147671686)	81.905	315
fig|6666666.230735.peg.629	VFG007298(gi:15642189)	81.864	397
fig|6666666.230735.peg.4177	VFG044440(gi:42491190)	81.847	314
fig|6666666.230735.peg.687	VFG007449(gi:147673876)	80.952	147
fig|6666666.230735.peg.614	VFG007388(gi:15642204)	80.851	141
fig|6666666.230735.peg.642	VFG013466(gi:68249928)	80.427	281
fig|6666666.230735.peg.2037	VFG007630(gi:37678524)	80.114	704

Table S5. Virulence factors identified in *V. fluvialis* 3663

Sequence ID	VFDB ID	identity (%)	alignment length
fig|6666666.230986.peg.436	VFG044042(gi:23663958)	98.243	740
fig|6666666.230986.peg.1395	VFG007568(gi:15642065)	96.825	126
fig|6666666.230986.peg.1393	VFG007580(gi:15642067)	96.61	295
fig|6666666.230986.peg.4133	VFG007352(gi:15642198)	96.377	138
fig|6666666.230986.peg.1401	VFG007544(gi:15642059)	96.341	164
fig|6666666.230986.peg.81	VFG007601(gi:27363787)	95.635	252
fig|6666666.230986.peg.2321	VFG007139(gi:147674396)	94.767	172
fig|6666666.230986.peg.821	VFG007138(gi:15600788)	94.03	134
fig|6666666.230986.peg.78	VFG007138(gi:15600788)	94.03	134
fig|6666666.230986.peg.4131	VFG007364(gi:15642200)	93.818	275
fig|6666666.230986.peg.729	VFG006908(gi:15642422)	93.204	103
fig|6666666.230986.peg.3398	VFG007466(gi:15642131)	93.003	343
fig|6666666.230986.peg.3396	VFG007454(gi:15642129)	92.694	438
fig|6666666.230986.peg.3268	VFG002093(gi:15600879)	92.668	491
fig|6666666.230986.peg.3391	VFG007424(gi:15642124)	92.647	136
fig|6666666.230986.peg.4400	VFG043132(gi:28900124)	92.337	261
fig|6666666.230986.peg.3392	VFG007430(gi:15642125)	92.264	349
fig|6666666.230986.peg.1394	VFG007574(gi:15642066)	92.213	244
fig|6666666.230986.peg.4137	VFG007328(gi:15642194)	91.985	262
fig|6666666.230986.peg.4128	VFG007382(gi:15642203)	91.837	49
fig|6666666.230986.peg.4132	VFG007358(gi:15642199)	91.603	131
fig|6666666.230986.peg.1391	VFG007594(gi:28899009)	91.559	699
fig|6666666.230986.peg.4126	VFG043121(gi:15642205)	91.538	130
fig|6666666.230986.peg.131	VFG018241(gi:15640579)	91.279	172

fig|6666666.230986.peg.4134	VFG007346(gi:15642197)	91.064	235
fig|6666666.230986.peg.4125	VFG043122(gi:15642206)	90.995	211
fig|6666666.230986.peg.3262	VFG002082(gi:15600885)	90.541	444
fig|6666666.230986.peg.2398	VFG007616(gi:15642596)	90.521	211
fig|6666666.230986.peg.4130	VFG007370(gi:15642201)	90.26	308
fig|6666666.230986.peg.4139	VFG007316(gi:15642192)	90.028	361
fig|6666666.230986.peg.4136	VFG007334(gi:15642195)	89.96	249
fig|6666666.230986.peg.4371	VFG043156(gi:28901401)	89.813	697
fig|6666666.230986.peg.3267	VFG002094(gi:15600880)	89.655	145
fig|6666666.230986.peg.610	VFG007659(gi:59710773)	89.174	351
fig|6666666.230986.peg.3154	VFG007087(gi:27364322)	89.116	147
fig|6666666.230986.peg.80	VFG007608(gi:37679056)	89.103	312
fig|6666666.230986.peg.3403	VFG007496(gi:15642136)	89.035	456
fig|6666666.230986.peg.3404	VFG007504(gi:28899028)	88.971	136
fig|6666666.230986.peg.689	VFG043143(gi:28901388)	88.789	446
fig|6666666.230986.peg.1957	VFG007661(gi:59710774)	88.614	202
fig|6666666.230986.peg.3269	VFG002092(gi:15600878)	88.485	165
fig|6666666.230986.peg.3409	VFG007532(gi:15642142)	88.329	377
fig|6666666.230986.peg.791	VFG042879(gi:27364897)	88.116	345
fig|6666666.230986.peg.4367	VFG043152(gi:28901397)	88.115	244
fig|6666666.230986.peg.3388	VFG007409(gi:27365291)	87.64	89
fig|6666666.230986.peg.1959	VFG007640(gi:37678525)	87.634	372
fig|6666666.230986.peg.4138	VFG007322(gi:15642193)	87.603	242
fig|6666666.230986.peg.3386	VFG007394(gi:15642119)	87.574	338
fig|6666666.230986.peg.4135	VFG007340(gi:15642196)	87.558	434
fig|6666666.230986.peg.3400	VFG007478(gi:15642133)	87.379	103

fig|6666666.230986.peg.4143	VFG007292(gi:15642187)	87.071	379
fig|6666666.230986.peg.116	VFG043148(gi:28901393)	86.907	443
fig|6666666.230986.peg.4404	VFG043128(gi:28900120)	86.806	144
fig|6666666.230986.peg.3260	VFG002084(gi:15600887)	86.782	870
fig|6666666.230986.peg.1972	VFG045346(gi:37679074)	86.617	269
fig|6666666.230986.peg.3399	VFG007472(gi:15642132)	86.575	581
fig|6666666.230986.peg.4368	VFG043153(gi:28901398)	86.517	89
fig|6666666.230986.peg.2524	VFG006965(gi:27364824)	86.237	574
fig|6666666.230986.peg.4402	VFG043130(gi:28900122)	86.181	398
fig|6666666.230986.peg.790	VFG042880(gi:27364898)	86.141	368
fig|6666666.230986.peg.3156	VFG007096(gi:15642726)	85.885	503
fig|6666666.230986.peg.4398	VFG043134(gi:28900126)	85.791	373
fig|6666666.230986.peg.3402	VFG007490(gi:15642135)	85.714	350
fig|6666666.230986.peg.3261	VFG002083(gi:15600886)	85.603	257
fig|6666666.230986.peg.3155	VFG007090(gi:15642725)	85.504	407
fig|6666666.230986.peg.3401	VFG007487(gi:27365278)	85.35	471
fig|6666666.230986.peg.3389	VFG007412(gi:15642122)	85.294	272
fig|6666666.230986.peg.3256	VFG002088(gi:15600891)	85.267	1181
fig|6666666.230986.peg.3157	VFG007103(gi:147674158)	85.251	678
fig|6666666.230986.peg.4401	VFG043131(gi:28900123)	85.185	243
fig|6666666.230986.peg.591	VFG007651(gi:15640289)	85.024	621
fig|6666666.230986.peg.3266	VFG002078(gi:15600881)	84.89	589
fig|6666666.230986.peg.3263	VFG002081(gi:15600884)	84.81	158
fig|6666666.230986.peg.1813	VFG007611(gi:147674163)	84.694	294
fig|6666666.230986.peg.4144	VFG007286(gi:15642186)	84.615	377
fig|6666666.230986.peg.1398	VFG007553(gi:27365301)	84.555	382

fig|6666666.230986.peg.4375	VFG043159(gi:28901406)	84.375	128
fig|6666666.230986.peg.3408	VFG007528(gi:28899032)	84.309	376
fig|6666666.230986.peg.3410	VFG007538(gi:15642143)	84.309	376
fig|6666666.230986.peg.686	VFG043146(gi:28901391)	84.231	520
fig|6666666.230986.peg.2174	VFG013418(gi:16273103)	84.211	190
fig|6666666.230986.peg.3952	VFG007245(gi:15600997)	83.601	311
fig|6666666.230986.peg.4369	VFG043154(gi:28901399)	83.333	258
fig|6666666.230986.peg.4405	VFG043127(gi:28900119)	83.333	120
fig|6666666.230986.peg.4380	VFG043164(gi:28901411)	83.158	285
fig|6666666.230986.peg.687	VFG043145(gi:28901390)	82.891	339
fig|6666666.230986.peg.4370	VFG043155(gi:28901400)	82.713	376
fig|6666666.230986.peg.3387	VFG007404(gi:37680653)	82.692	260
fig|6666666.230986.peg.1392	VFG007586(gi:15642068)	82.54	504
fig|6666666.230986.peg.609	VFG007661(gi:59710774)	82.517	286
fig|6666666.230986.peg.4127	VFG007388(gi:15642204)	82.27	141
fig|6666666.230986.peg.2473	VFG006902(gi:15642421)	82.206	562
fig|6666666.230986.peg.4142	VFG007298(gi:15642189)	81.864	397
fig|6666666.230986.peg.3648	VFG044440(gi:42491190)	81.847	314
fig|6666666.230986.peg.3654	VFG044437(gi:42491184)	81.818	176
fig|6666666.230986.peg.688	VFG043144(gi:28901389)	81.59	239
fig|6666666.230986.peg.3265	VFG007117(gi:147671686)	81.587	315
fig|6666666.230986.peg.3395	VFG007449(gi:147673876)	80.952	147
fig|6666666.230986.peg.1961	VFG007627(gi:37678523)	80.328	61
fig|6666666.230986.peg.1960	VFG007630(gi:37678524)	80.114	704
fig|6666666.230986.peg.1360	VFG013466(gi:68249928)	80.071	281

Table S6. Summary of prophage regions in *V. fluvialis* strain 12605 and 3663

Strain	Region	Region length/kb	Completeness	CDS	Specific keyword
12605	1	7.9	intact	10	coat
	2	10.1	intact	14	coat
	3	50.2	intact	48	tail,lysin,head,terminase,capsid,portal,integrase
	4	13.5	incomplete	16	tail,capsid,portal
	5	24.6	questionable	24	tail,plate,integrase
3663	1	28.1	questionable	33	portal,tail,plate,integrase
	2	17.1	questionable	9	transposase,tail,integrase
	3	42	incomplete	32	integrase,terminase,head,portal
	4	10.1	intact	14	coat

Table S7. The ortholog gene statistics of *V. fluvialis* strains

Strains	Total CDSs	Specific Genes of Strains	Core Genes	Core genes/Total CDSs	Core/Pan genes
12605	4395	1248		28%	
3663	4441	1294		29%	
ATCC 33809	4406	1259		29%	
PG41	4845	1698		35%	
I21563	4050	903	3147	22%	41.3%
560	4364	1217		28%	
539	5609	2462		44%	
NBRC 103150	4373	1226		28%	
S1110	4129	982		24%	

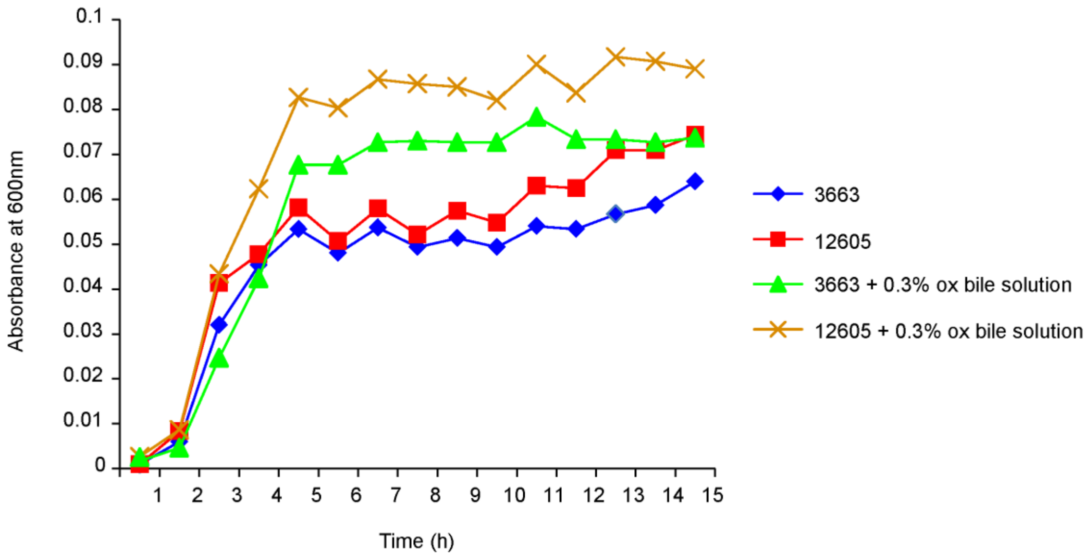

Figures S1. Growth curve of two *V. fluvialis* strains

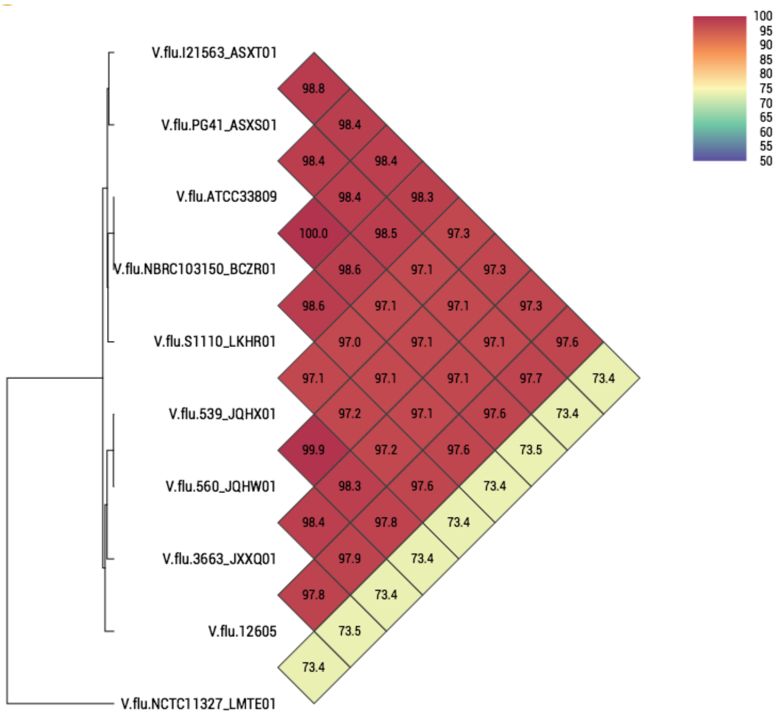

Figure S2. ANI genomic comparison of ten *V. fluvialis* strains

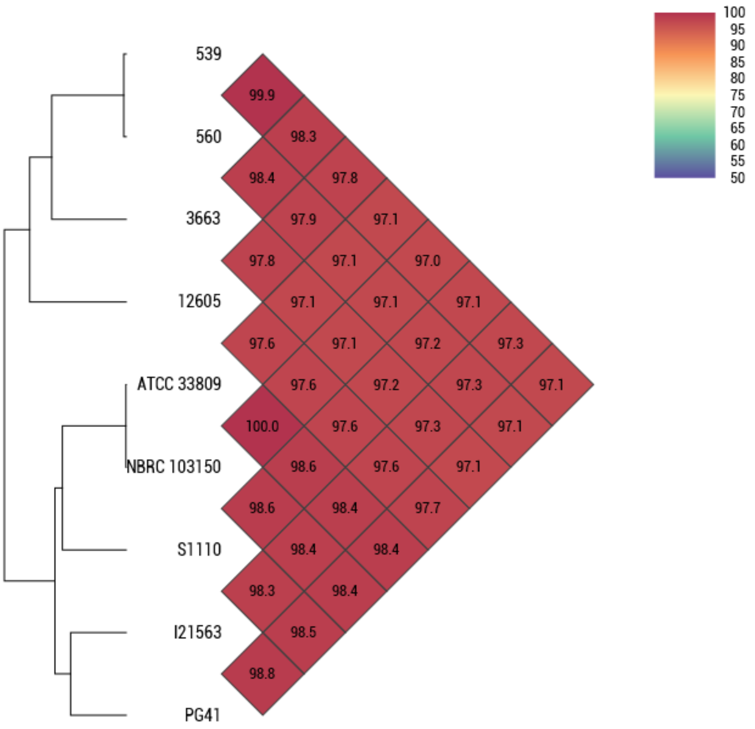

Figure S3. ANI genomic comparison of nine *V. fluvialis* strains

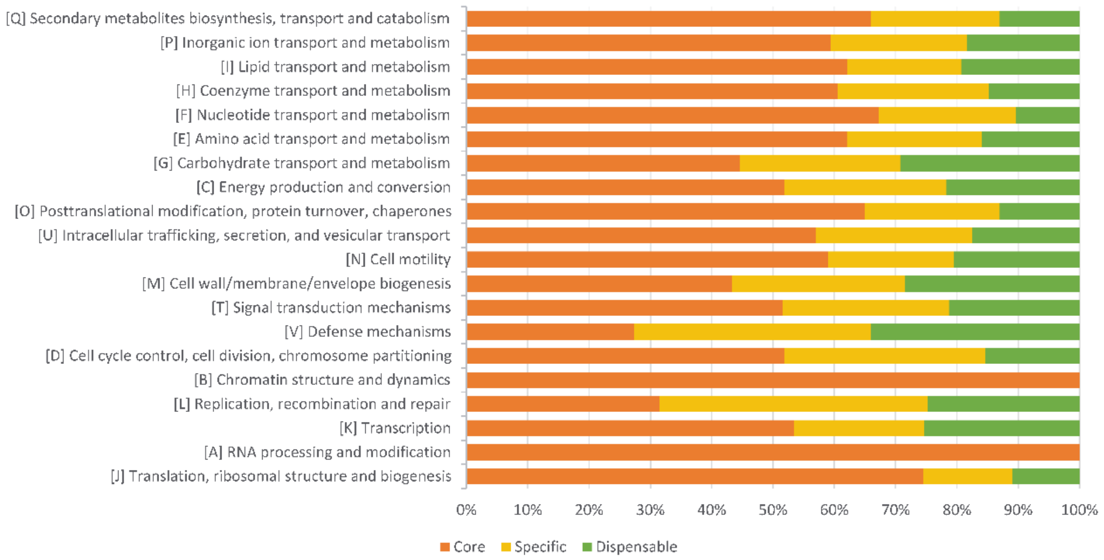

Figure S4. Distribution of orthologous genes based on COG categories
